# Unraveling genome- and immunome-wide genetic diversity in modern and historical Jaguars

**DOI:** 10.1186/s13059-025-03868-0

**Published:** 2025-12-08

**Authors:** René Meißner, Sven Winter, Jean Pierre Elbers, Martin Plášil, Ján Futas, Elmira Mohandesan, Muhammad Bilal Sharif, Petr Horin, Stefan Prost, Pamela Burger

**Affiliations:** 1 Research Institute of Wildlife Ecology, University of Veterinary Medicine Vienna, Vienna, Austria; 2 Faculty of Science and Technology, University of the Faroe Islands, Tórshavn, Faroe Islands; 3 Institute of Medical Genetics, Center for Pathobiochemistry and Genetics, Medical University of Vienna, Vienna, Austria; 4 Department of Animal Genetics, University of Veterinary Sciences, Brno, Czechia Czechia; 5 Research Group Animal Immunogenomics, CEITEC Vetuni, Brno, Czechia Czechia; 6 Department of Evolutionary Anthropology, University of Vienna, Vienna, Austria; 7 Human Evolution and Archaeological Sciences (HEAS), University of Vienna, Vienna, Austria; 8 Konrad Lorenz Institute of Ethology, University of Veterinary Medicine Vienna, Vienna, Austria; 9 Centre for Palaeogenetics, Stockholm, Sweden; 10 Ecology and Genetics Research Unit, University of Oulu, Oulu, Finland; 11 Natural History Museum Vienna, Central Research Laboratories, Vienna, Austria; 12 South African National Biodiversity Institute, National Zoological Garden, Pretoria, South Africa

**Keywords:** Jaguar conservation, Genetic diversity, Population structure, Evolutionarily significant units, Immune response genes, NKC, TLR, MHC

## Abstract

**Background:**

The jaguar (*Panthera onca*) is a keystone species within diverse ecosystems ranging from dense rainforests to open grasslands across Central and South America. However, its populations are declining rapidly due to anthropogenic actions, such as deforestation and poaching. Here we investigate the effects of this decline on genetic diversity and genetic health. Utilizing both modern and historical museum samples, we infer population structure and immunome variability in 25 jaguars to identify unique genetic diversity that can inform targeted conservation efforts.

**Results:**

Our genome-wide analyses identifies three distinct geographic populations: Central America, South American lowlands, and South American highlands. Modern samples that exhibit lower levels of heterozygosity also show higher levels of inbreeding. The South American lowland population shows the lowest levels of inbreeding, while the highland population exhibits the lowest overall immunome-wide variability. However, the innate (Natural Killer Cell Complex, Toll-Like Receptor) and adaptive (Major Histocompatibility Complex Class II) immune genes, which are crucial for adaptive responses and disease resilience, show high diversity in terms of heterozygosity and haplotype diversity in individuals of all three populations.

**Conclusions:**

South American highland and Central American jaguars face significant threats from habitat loss and fragmentation. The observed genome- and immunome-wide diversity in historical and modern jaguars reflect their recent demographic decline and challenges of local adaptation. We recommend re-evaluating evolutionarily significant units to prioritize conservation strategies, ensuring the preservation of unique genetic and adaptive diversity crucial for the species’ resilience and long-term survival.

**Supplementary Information:**

The online version contains supplementary material available at 10.1186/s13059-025-03868-0.

## Background

The jaguar (*Panthera onca*) stands out as one of the most distinctive members of the genus *Panthera* and is America’s largest feline predator [1]. Unusual for its genus, the jaguar is very muscular for its size and possesses the strongest bite of all extant felids [2]. The species’ remarkable strength is also reflected in its specialized hunting method, which consists of jumping on top of its prey, followed by a fatal bite through the skull [3]. Capable of hunting even the largest mammals, jaguars are not bound to specific habitats, further consolidating their position as apex predators [4]. Consequently, they are a keystone species within diverse ecosystems ranging from dense rainforests to open grasslands across Central and South America [5].

Despite their broad distribution and ecological importance, jaguar populations are highly threatened by deforestation resulting from human population growth and intensifying agriculture [6]. Throughout the Americas, this increasing habitat loss is pressurizing wildlife. Furthermore, jaguars face additional anthropogenic threats like poaching, persecution, and habitat fragmentation, which more severely threaten large carnivores with extensive territories than their prey’s populations [7]. For instance, a recent increase in human-made wildfires in Bolivia and Brazil has led to the obliteration of 12% of the Chiquitano Dry Forest [8], which threatens the resident jaguar population in an already shrinking but important wildlife corridor [9]. Several parts of Brazil already exhibit high levels of habitat fragmentation that are responsible for local extinction, and thus reduced gene flow is increasingly threatening the remnant jaguar populations [10]. Furthermore, the poaching of jaguars has increased drastically over recent years because the traditional medicine market in Asia discovered jaguar fangs as a suitable substitute for decreasingly available tiger fangs [11, 12]. Climate change intensifies these challenges, further increasing habitat degradation, altering prey availability, and escalating the frequency of extreme weather events. However, the jaguar as a species is only listed as “Near Threatened” by the IUCN with no current subspecies assignment, although increased regional population decline is acknowledged, particularly in Central America [13].

A taxonomic re-evaluation of the species based on morphological data [14] and an extensive microsatellite study [15] prompted the IUCN to discard the previously assigned nine subspecies [13]. Because no current subspecies are recognized, the misconception that individual population losses in the jaguar are less severe than in other large carnivores prevails [16]. Yet, populations are increasingly declining, and the identification of evolutionarily significant units (ESUs) in the species is urgent to assist conservation planning and management [17, 18]. When different populations in a species exhibit high degrees of genetic differentiation, ESUs should be assigned to reflect potential adaptational differences in the species subpopulation [19]. Furthermore, recent studies using an updated set of microsatellites found noticeable genetic structure within jaguars [20] and, combined with an initial whole-genome study [21], supported a pronounced distinction between the South and Central American populations. However, the degree to which this genetic distinction affects the jaguar’s adaptive potential in its habitats is yet completely unknown.

Generally, a species’ adaptability is difficult to assess directly because it involves complex interactions between genetic, physiological, ecological, and behavioral factors, which are challenging to measure comprehensively and simultaneously [22]. Nevertheless, several proxies exist that can serve as estimates for an organism’s resilience to environmental changes. Genetic diversity is one of the most reliable estimators because high genetic diversity within a population relates to a higher likelihood of adaptive responses to selective pressures from changing environments [23]. The genetic diversity in immune response genes is directly translated into amino acid sequences, hence, accessing genetic diversity is more closely tied to the immediate immune function [24]. Increased genetic diversity in immune response genes correlates with the ability to recognize and respond to a broad spectrum of pathogens, therefore increasing a species’ resilience to diseases [25].

The recognition of pathogens is essential to activating innate immune response, and specific pattern recognition receptors, such as Toll-like receptors (TLRs) and receptors of the natural killer cell complex, induce this activation [26]. For instance, the Killer cell lectin-like receptors (KLR) of the natural killer cell complex recognize specific molecules on cells, endogenous and non-endogenous, either activating natural killer cells upon ligand binding or inhibiting natural killer cell activity when engaging with self-MHC class I molecules [27]. TLRs, on the other hand, detect a wide array of foreign microbial structures known as pathogen-associated molecular patterns, which can be bacterial cell wall components or virus-associated RNA [28]. All these receptors exhibit structural variability and possess high diversity in their ligand recognition domains. Averting diseases is crucial for survival, therefore, assessing immunity, especially innate immune response genes, can serve as an indicator of a species’ adaptive potential [29]. Few studies acknowledge this connection and focus on immune response genes and their implications for adaptability, especially in wild felids.

In this study, we combined immune genetic and population genomic approaches to shed light on the sparsely understood population structure of the jaguar using contemporary and historical data. We further compared genome-wide diversity to that of the innate and adaptive immune response genes in light of the observed population structure and genetic health. Our sampling encompassed large portions of the present-day distribution of the species to assist in the re-evaluation of the jaguar’s systematics. We generated whole-genome data for both contemporary and historical individuals and also incorporated individuals from an already existing dataset [21].

Furthermore, we examined the immunome and investigated the diversity of three distinct immune response gene families in contemporary jaguars: TLR, NKC, and MHC class II. With that, we aimed to enhance our understanding of important immune response genes underlying the species’ disease resilience and adaptive potential. Despite habitat degradation impacting genetic diversity and structure in jaguar populations [30], there has not been any assessment of the species’ immune response genes to evaluate its adaptive potential.

## Results

This study investigated population structure in “Near Threatened” Central and South America jaguars to aid in establishing ESUs and to evaluate the species’ adaptive potential by generating comprehensive whole-genome- and immunome-wide data for 11 historical and 14 modern individuals. We examined population structure in jaguars using genome- and immunome-wide SNP data to validate the assumed population structure based on ecoregions and investigate the distinction between individual populations. Sufficient genetic distinction is an important criterion for assessing ESUs in a species; therefore, we further examined diversity parameters such as nucleotide diversity, inbreeding, and heterozygosity and measured pairwise fixation indices between all jaguar populations. Additionally, the genetic diversity of two innate (TLR, NKC) and one adaptive (MHC class II) immune response gene families were examined to obtain an approximation of the species’ adaptive potential.

### Genome- and immuno-wide SNP data reveal three distinct Jaguar populations

**Fig. 1:**
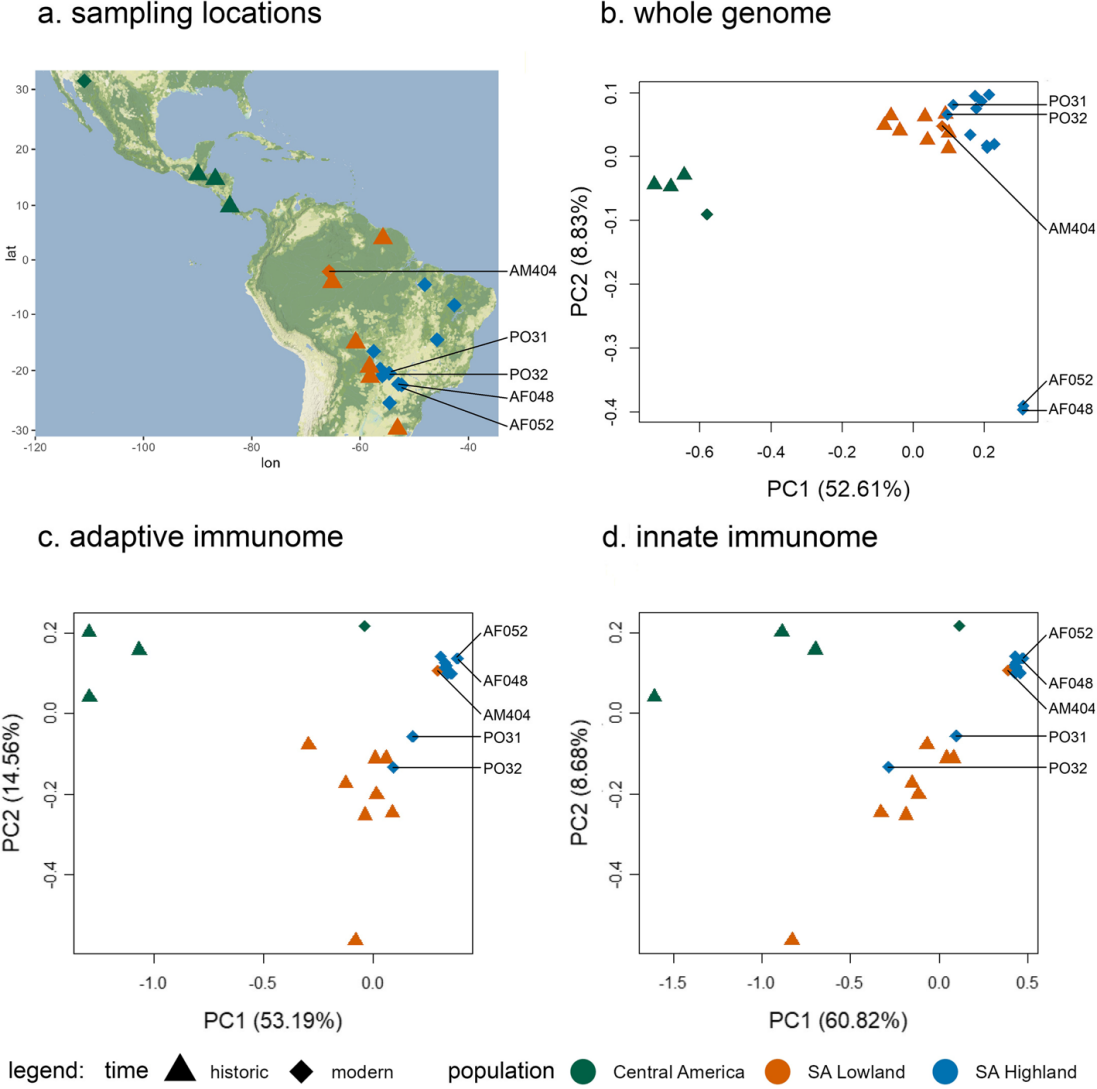
(**a**) Approximate sampling locations for all 25 jaguar individuals used in this study. Principal component analysis showing the first two principal components based on 689,785 unlinked genome-wide SNPs (**b**), based on 14,155 unlinked adaptive immunome-wide SNPs (**c**), and based on 26,408 unlinked innate immunome-wide SNPs (**d**) of 25 jaguar individuals

The genome-wide analysis of 689,785 unlinked SNPs from 25 individuals showed that jaguars can be separated into different geographic populations across their distribution. The PCA revealed a clear separation into three clusters (Fig. 1b), corresponding to three distinct regions: Central America, South American lowlands, and South American highlands. The Central American jaguars are separated by PC1 (28.52%) from the South American individuals, while the two clusters of South American highland and lowland individuals are separated by PC2 (9.05%) and PC3 (7.26%). Two South American highland individuals, PO31 and PO32, clustered together with the South American lowland population, while one individual of the South American lowland population, AM404, grouped within the South American highland population. Within the cluster of the South American highland population, two individuals, AF048 and AF052, were distant from all other individuals but grouped close to each other.

Separately conducted PCAs using adaptive (14,155 SNPs) and innate (26,408 SNPs) immunome data generally exhibited less pronounced clustering of jaguar populations than the PCA based on genome-wide SNPs. However, all three clusters were discernible (Fig. 1b-d). Central American jaguar individuals were distinguished from South American jaguars by PC1 (53.19% adaptive, 60.82% innate), while PC2 (14.56% adaptive, 8.68% innate) separated the South American lowland and highland populations. PC3 of the adaptive and innate immunome explained less than 5% of the observed variation (Additional file 1: Fig. S1). However, unlike the PCA based on genome-wide SNPs (Fig. 1b, Additional file 1: Fig. S2), the within-cluster variation of adaptive and innate immunome as well as that of the exome was comparatively lower in the South American highland population than in both other populations (Additional file 1: Fig. S3). Notably, the individuals AF048 and AF052, contrary to their clustering in the PCA based on genome-wide SNPs, did not group distant to other individuals from the South American lowland population. Furthermore, the modern individual MDAZ grouped closer with individuals from the South American populations in both immunome-wide PCAs than with individuals from the Central American population, unlike in the PCA based on genome-wide SNPs.

The highest population differentiation was observed between Central America and the South American highland (F_st_ = 0.18). In contrast, the differentiation between Central America and the South American lowland (F_st_ = 0.065) and between the South American lowland and highland population (F_st_ = 0.042) were lower. Considering both South American lowland and highland individuals belonging to a single population, the differentiation between this unified South American population and the Central American population was slightly lower (Fst = 0.134) than between Central America and the South American highlands.

**Fig. 2:**
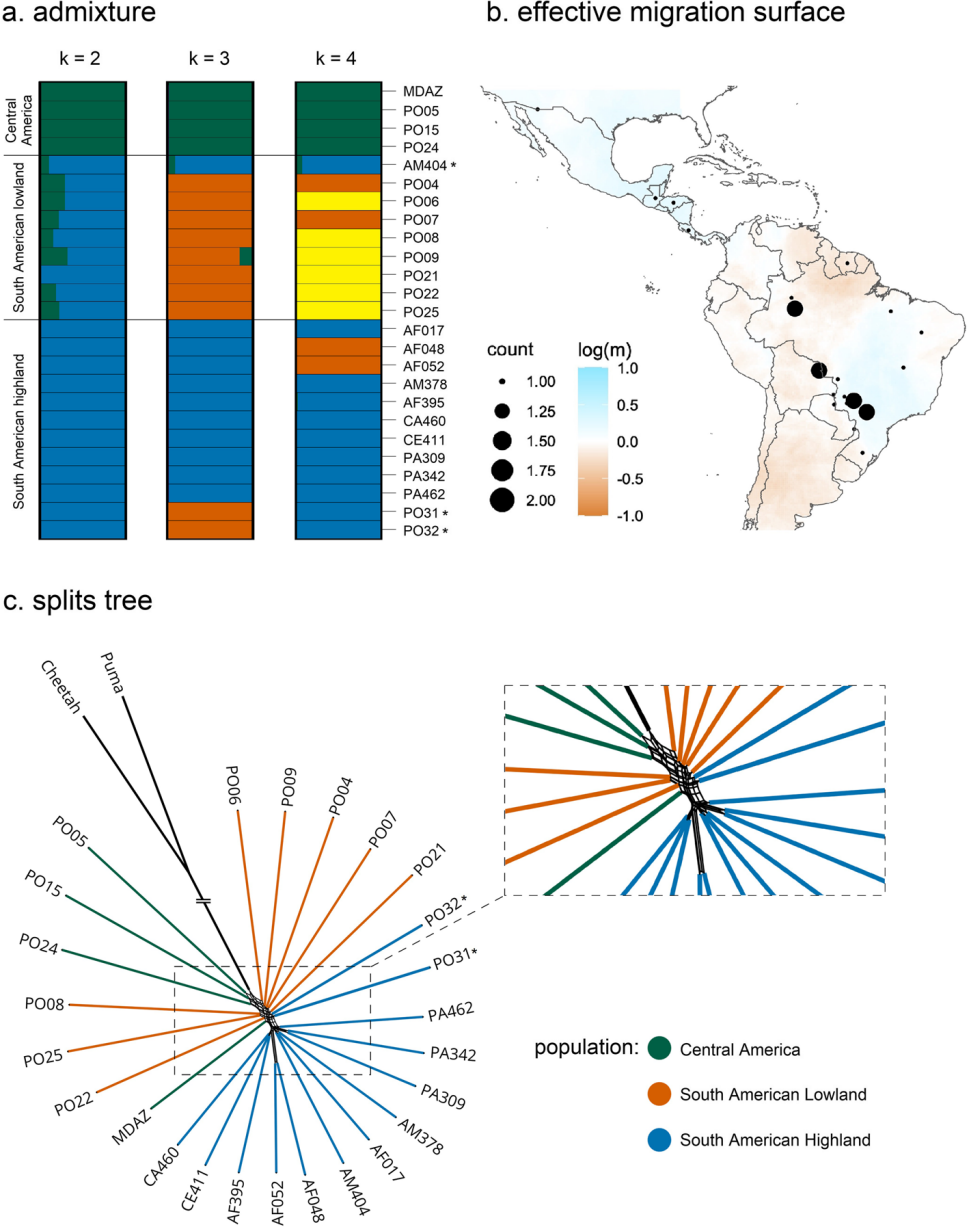
(**a**) Admixture analysis of 689,785 unlinked genome-wide SNPs with K ranging from 2 to 4. (**b**) Effective migration surface for all 25 jaguar individuals supporting migration within the Central and South America highland population but not within the South American lowland population, blue indicates higher than overall average migration, white corresponds to the overall mean migration, and brown indicates strong barriers to migration. (**c**) Phylogenetic relationships of the 25 jaguar individuals inferred by the phylogenetic network approach implemented in SplitsTree, asterisks indicated samples that were potentially assigned to the wrong population due to unclear sampling location

Using the same unlinked SNPs of the genome-wide PCA, the admixture analysis reflected the previously detected population structure and revealed limited genetic exchange between the three distinct jaguar populations (Fig. 2a). At K = 2, the Central American population was separated from both South American populations, with some degrees of admixture present in the lowland population. At K = 3, the South American lowland and highland populations were further segregated. According to the variance in log likelihoods and the pairwise correlation of residuals from the evalAdmix analyses, the underlying population structure is largely resolved at K = 3 or higher (Additional file 1: Fig. S4 and S5). The same individuals (AM404, PO31, PO32) that deviated in the PCAs from their assumed populations based on geographic origin are also evident here. For K > 3, no further population structure is apparent that can be correlated with geographical patterns in jaguars. Noticeably at K = 4, the individuals AF048 and AF052 from the South American highland population, which clustered distantly to their assigned population based on origin in the whole genome PCA, grouped with two individuals from the South American lowland population, PO04 and PO07, without any apparent connection to a geographic pattern.

Furthermore, we examined migration between jaguar populations by estimating the effective migration surface for all 25 individuals (Fig. 2b). Higher than average migration was observed within the Central American and South American highland populations, and lower than average migration was observed within the South American lowland population. However, the lower-than-average migration could also be an artifact of the limited sample size with known GPS coordinates for this region.

Within the phylogenetic network constructed from whole-genome data of 25 jaguar individuals, three separate clusters are recovered reflecting two populations in South America and one in Central America (Fig. 2c). Notably, MDAZ deviates from its assigned population based on origin, clustering with the South American lowland populations rather than the Central American population (Figs. 1a and 2c).

### Genome-wide and immunome nucleotide diversity between Jaguar populations

**Fig. 3:**
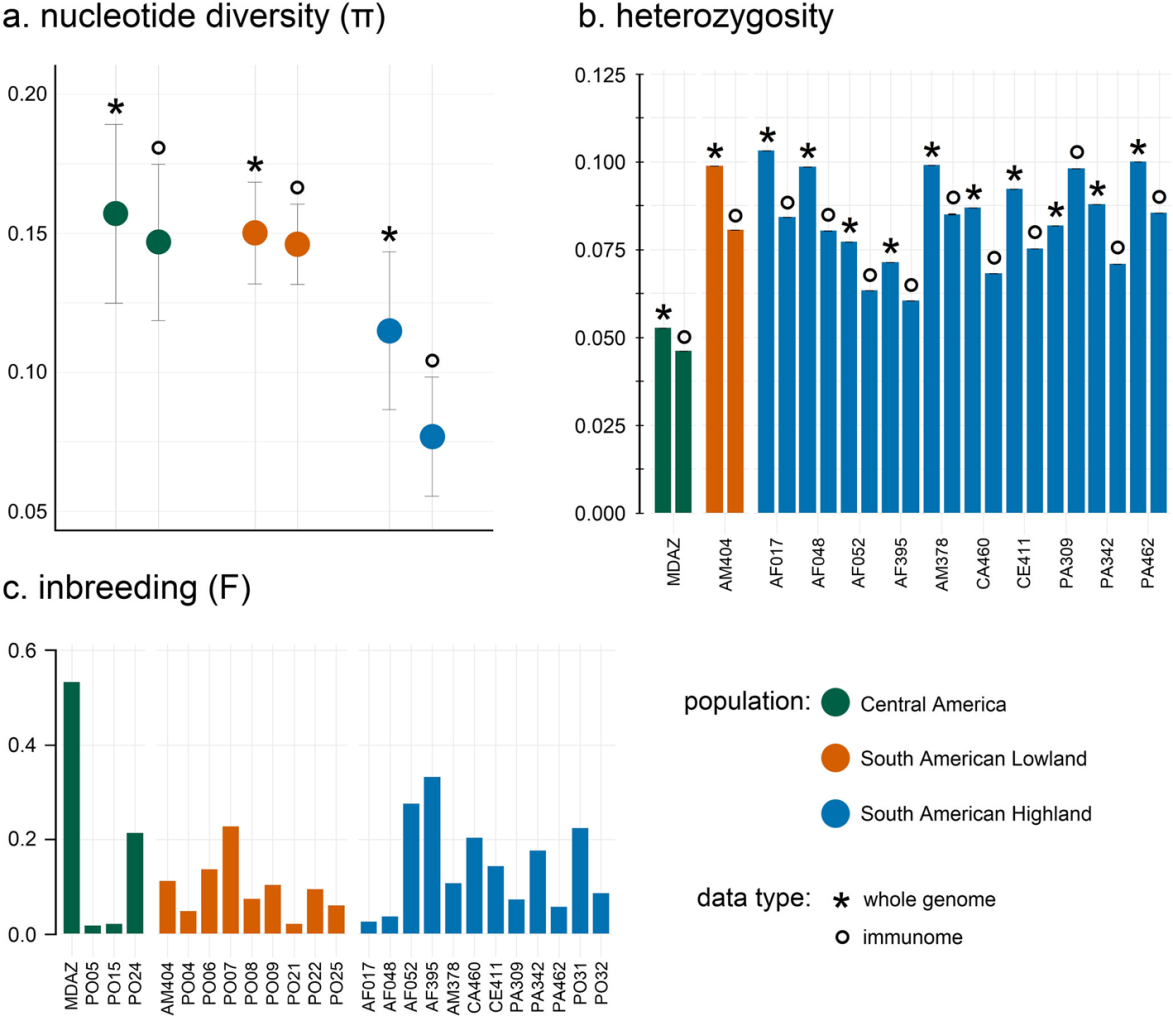
(**a**) Mean nucleotide diversity [π] across the whole genome and immunome for three jaguar populations, with error bars representing the standard deviation. (**b**) Individual-level heterozygosity across the whole genome and immunome for modern jaguars (excluding PO31 and PO32). (**c**) Individual genome-wide inbreeding coefficient [F] for all jaguar samples. Populations are color-coded: green = Central America, orange = South American lowland, and blue = South American highland. Data type is distinguished by markers: asterisks (*) indicate whole-genome estimates, and circles (o) represent immunome estimates.

Nucleotide diversity (π) across the three jaguar populations ranged from 0.115 to 0.157 in the whole genome and from 0.077 to 0.147 in the immunome (Fig. 3a). The highest genome-wide diversity (π = 0.157) was observed in the Central American population, while the lowest (π = 0.115) occurred in the South American highland population. The lowest immunome-wide nucleotide diversity (π = 0.076) was also observed in the highland population, whereas the highest (π = 0.147) was found in the Central American population. In both the Central American and South American lowland populations, genome-wide and immunome-wide diversity values were relatively similar. By contrast, the South American highland population showed a more pronounced reduction in immunome diversity compared to genome-wide estimates. Differences in nucleotide diversity between whole-genome, complete immunome, adaptive, and innate immune gene sets were generally minor, except in the highland population, where immune-related regions exhibited consistently lower diversity than that of the whole genome (Additional file 2: Table S1). Both the whole-genome and immunome datasets showed significant differences (*p* ≤ 0.0001) in genetic diversity among the three populations based on the distribution of per-site nucleotide diversity (Additional file 1: Fig. S7). However, because the sample size for Central American jaguars was small (*n* = 4), these results should be interpreted cautiously.

The genome-wide and immunome-wide heterozygosity of 12 modern jaguar individuals (excluding PO31 and PO32 due to insufficient coverage) ranged between 0.10% and 0.04% (Fig. 3b). The lowest values were observed in MDAZ from the Central American population (whole genome = 0.053%, immunome = 0.046%) and two individuals, AF052 (whole genome = 0.077%, immunome = 0.063%) and AF395 (whole genome = 0.071%, immunome = 0.061%), from the South American highland population. The genome-wide (0.053%) and immunome-wide (0.046%) heterozygosity of MDAZ was almost half of the values observed in some South American highland individuals, e.g., AF017 (whole genome = 0.103%) and PA309 (immunome = 0.092%). The heterozygosity of the immunome was consistently lower than that of the whole genome, except for individual PA309, where the heterozygosity of the immunome was higher. The levels of genome-wide inbreeding per individual varied between historical and modern samples, with the inbreeding coefficient ranging from 0.019 to 0.227 in historical samples, and from 0.027 to 0.533 in modern samples (Fig. 3c). Modern samples exhibited low levels of heterozygosity and showed high levels of inbreeding. The population with the lowest levels of inbreeding was the South American lowland population. The inbreeding coefficients calculated by runs of homozygosity for the contemporary individuals in Lorenzana et al. (2021) are comparable to our ngsF estimates.

It is worth noting that our sampling did include both modern and historical samples, which could impact genomic inferences. However, given the low expected damage rate given the age of the samples [31] we do not expect this to impact the analyses strongly. Indeed, we only observed very minor differences when running the analyses on transversions only (data not shown), as transitions can potentially be impacted by DNA damage in historical samples [32]. This indicates that the results are not driven by damage pattern in the historical samples.

### Specific innate and adaptive immune response gene diversity

We were able to examine nine genes of the NKC, all ten TLR genes expected in felids, and two MHC class II genes in the 12 modern jaguar samples, which showed sufficiently high coverage (> 10x, Additional file 2: Table S2) to call heterozygous alleles reliably. These samples included ten individuals from the South American highlands and one sample each of the South American lowlands and Central American population, respectively, which have also been used for the heterozygosity estimation. The genes belonging to the NKC included KLRA, six KLRC genes: four members of KLRC1 (KLRC1-1, KLRC1-4, KLRC1-5, KLRC1-6), KLRC2, KLRC3, KLRH4, and KLRJ. TLR genes included TLR1-10, and MHC class II genes included DMA and DRA. The nuclear allele count ranged between 2 and 23 and was higher or equal to the number of resulting amino acid sequences that ranged from 1 to 23 (Table 1). Most genes, irrespective of their family, exhibited high heterozygosity (≥ 0.5) and haplotype diversity (≥ 0.6), with only a few genes falling below these values. Notably, both TLR3 and TLR8 exhibited heterozygosity below 0.01 and haplotype diversity below 0.4. KLRA and DMA displayed the lowest number of nuclear alleles (N_alleles_ = 2), and TLR7 and TLR8 showed the lowest number of amino acid sequences (N_amino acid_ = 1). The lowest heterozygosity was observed in TLR3 and TLR8 (H = 0.083), which also showed the lowest haplotype diversity (h = 0.3254). For several genes (TLR2, TLR6, DRA), all jaguar individuals carried two different full-length alleles, with no individuals being homozygous for a single haplotype.

**Table 1:** Comparison of innate (NKC, TLR) and adaptive (MHC class II) immune response gene diversity within 12 modern jaguars (excluding PO31 and PO32)

gene family	gene	Nalleles	Namino acid	H	h
NKC	KLRA	2	2	0.9167	0.5181
NKC	KLRC1-1	7	6	0.5833	0.6957
NKC	KLRC1-4	16	16	0.3333	0.9710
NKC	KLRC1-5	23	21	0.9167	0.9964
NKC	KLRC1-6	23	23	0.9167	0.9964
NKC	KLRC2	10	5	0.1667	0.8913
NKC	KLRC3	4	4	0.5833	0.5435
NKC	KLRH4	12	12	0.75	0.9130
NKC	KLRJ	5	5	0.4167	0.6268
TLR	TLR1	15	10	0.5	0.9286
TLR	TLR2	17	12	1	0.9550
TLR	TLR3	3	2	0.083	0.2037
TLR	TLR4	8	4	0.5833	0.7434
TLR	TLR5	11	7	0.75	0.8360
TLR	TLR6	9	6	1	0.8360
TLR	TLR7	6	1	0.25	0.7196
TLR	TLR8	4	1	0.083	0.3254
TLR	TLR9	12	7	0.75	0.8730
TLR	TLR10	7	6	0.5	0.8069
MHC class II	DMA	2	2	0.5	0.5072
MHC class II	DRA	12	11	1	0.8841

## Discussion

### Population and immune genomic implications for conservation

Species lacking the assignment of ESUs, reflecting their evolutionary history and genetic distinction, give the impression of genetic uniformity. This lack poses a potential problem if it leads to an oversight of regional population decline that threatens unique and local genetic diversity [33]. The jaguar is currently without ESU or subspecies assignments, regardless of its large continent-spanning distribution. Consequently, the whole species is listed as “Near Threatened” by the IUCN as regional population decline is not sufficiently reflected in the most recent assignment [13]. However, jaguars are more severely endangered in certain parts of their distribution than the range-wide census population size estimates indicate [16], and their unique genetic diversity might already be threatened in regions of increased population decline.

Earlier microsatellite studies investigating population structure in jaguars did not detect noticeable genetic separation within the species [15]. However, our genome- and immunome-wide analyses support a genetic differentiation corresponding to three geographical regions in South and Central America. The separation between Central and South American jaguar populations has already been proposed using modern whole-genome data, but the sampling was not sufficient to make a reliable statement about delineation within South America [21]. Our genomic data additionally incorporated historical individuals from an expanded sampling area and supported not only the distinction between Central and South America but also separated at least two groups within South America. Furthermore, recent genetic studies employing different sets of microsatellites revealed more population structure in Central American [20, 34] and South American [35] jaguars than previously thought. However, we have to caution that there are large geographic regions between our Central and South American individuals that have not been sampled, and we cannot rule out a continuous population divergence between the two regions.

 While microsatellites support a separation into three distinct populations in South America, namely Amazon, Pantanal, and Atlantic Forest, our genomic data only suggested two: a South American lowland population including the rainforest of the Amazon, Pantanal, and Gran Chaco, and a South American highland population including the biomes of the Cerrado, Caatinga, and Atlantic Forest. Contrasting the microsatellite-based separation, whole-genome data did not identify a separate Pantanal population. This could be attributed to the higher power of genomic SNPs to identify distinct groups in clustering methods, compared to microsatellite data, which tends to overestimate local distinction [36, 37], or to our limited sampling in the area compared to the microsatellite studies [35]. Overall, the identified genetic structure in jaguars was similar to earlier studies, independent of the used population genetic markers. Nevertheless, SNPs are more precise than microsatellites in population-level diversity estimation and allow for additional adaptability assessments [36]. Therefore, we only considered the following three distinct jaguar populations based on whole-genome data: Central America, South American lowlands, and South American highlands.

While most jaguar individuals were correctly assigned to the three populations based on geographic ecoregions, three samples showed conflicting signals between genomic and geographic population assignments. Unlike other individuals from the Amazon, AM404 did not cluster with the South American lowland population but with the South American highland population, which might be explained by limited sampling in the region. So far, no genomic data for individuals from Central South America exist; thus, it is still unclear where precisely the boundary lies between the South American lowland and highland populations. Therefore, AM404 could potentially originate from the border of both populations, which led to its misclassification. Comparably, the individuals PO31 and PO32 could also be falsely assigned to the South American highland population based on their assumed geographic origin. The exact sampling locality for both individuals is unknown, but they originate from the Brazilian state of Mato Grosso do Sul. This state encompasses two different ecoregions, Pantanal and Cerrado, which belong either to the South American lowland population or to the South American highland population. As the Cerrado covers most of the state’s area, we initially assigned PO31 and PO32 to the South American highland population based on probability. However, using whole genome data, both individuals cluster with other samples of the South American lowland population and, therefore, are more likely to originate from the Pantanal. To assist sample assignments in the future, extending the sampling area, especially in regions where both South American jaguar populations border each other, is necessary, and the exact sampling locality must be noted. Yet, geographic ecoregion and genomic data, for the most part, agreed on the assignment of individual samples to the three distinct populations.

Despite their clear separation, jaguars seemed less strongly divergent than other big cats [38, 39]. The highest genetic distinction among jaguars was observed between the Central American and the South American highland populations (F_st_ = 0.18), which are also the most geographically distant. Conversely, the lowest genetic differentiation was identified between the South American lowland and South American highland populations (F_st_ = 0.04), which are geographically adjacent to each other. Considering both South American lowland and highland jaguars as part of a single population due to their moderate level of distinction, the differentiation between South and Central America was still high (F_st_ = 0.13). In comparison, recent studies based on genome-wide data identified F_st_ values ranging from 0.1 to 0.4 between subspecies of tigers (*Panthera tigris* [40]), and from 0.2 to 0.5 between subspecies of cheetahs (*Acinonyx jubatus* [41]),. Similar to [21], our findings show stronger separation between Central and South American jaguars, which falls within the range of F_st_ values reported for subspecies for other *Panthera* species (such as the tiger) and could justify classifying them as different ESUs or even subspecies [42, 43]. Based on our findings, re-assessing jaguar ESU is urgent to acknowledge the species’ genetic distinction and assist differentiated jaguar conservation efforts [18] 

The Central American and South American highland populations represent the northern and easternmost jaguar populations and are on the edge of the species’ range [16]. Therefore, these populations are more severely threatened by habitat loss because any level of habitat fragmentation is more likely to result in genetic isolation and impoverishment compared to the core population [44]. The most substantial threat to the jaguar, deforestation for land development purposes, is already causing intense habitat fragmentation in Central America [34] and limited gene flow between populations in the South American highlands 

Furthermore, two highland individuals, AF048 and AF052, already showed increased levels of inbreeding [21], and at least AF052 also exhibited decreased levels of heterozygosity. Similarly, increased inbreeding was observed for several subpopulations of Central American jaguars [38], and compared to both South American populations, the Central American population was even more genetically depauperate [20]. Our results further support these findings, as the single contemporary Central American jaguar in our study, MDAZ, was the least heterozygous individual within all contemporary individuals for which we were able to calculate heterozygosity, including South American lowland and highland populations. Nevertheless, modern individuals in both populations, Central America and South American highlands, show some reduction in genetic diversity and increased inbreeding. Thus, their adaptive potential might already be lowered compared to the South American lowland population.

### Immune genetic diversity to infer the adaptive potential in jaguars

To investigate the adaptive potential of the three jaguar populations, we looked in-depth into the species’ immunome, as it represents the entirety of annotated immune genes and is believed to consist mostly of genes under selection [39]. We discovered that the immunome mainly reflected the whole genome’s population structure in jaguars, but the within-population variability was noticeably reduced in the principal component analysis of the South American highland population.

Comparing nucleotide diversity between the adaptive and innate immunome regions, we observed minimal differences across all populations (Additional file 2: Table S1). In both the Central American and South American lowland populations, immunome-wide nucleotide diversity was similar to or slightly lower than whole-genome and exome estimates, consistent with the effects of purifying selection acting on immune-related genes [45]. In contrast, the South American highland population exhibited noticeably lower nucleotide diversity in both the exome and immunome compared to the other two populations, with the immunome diversity being particularly reduced, indicating strong genetic drift or bottlenecks, particularly affecting immune diversity [46]. The South American highland jaguars inhabit highly fragmented habitats with increased human pressure and disrupted connectivity, which enhances the effects of drift by reducing effective population size [20]. While increased exposure to novel pathogens through contact with livestock might be expected to promote immunogenetic diversity, the observed depletion suggests that demographic collapse has overwhelmed potential adaptive response [10]. Therefore, immune gene diversity in the South American highland population could not be elevated but instead reduced in parallel with genome-wide diversity, underscoring the disproportionate impact of habitat fragmentation and population decline. This emphasizes the need to monitor both genome-wide and immunome-specific diversity to adequately assess adaptive potential in fragmented and endangered wildlife populations [45, 47].

However, additional investigations into NKC, TLR, and MHC class II genes in the less genetically variable highland population did not reveal genetic impoverishment. In particular, KLRC1 genes were highly variable and exhibited increased levels of heterozygosity and haplotype diversity compared to other mammals, including camels [48], lemurs [49], and humans [50]. When examining the diversity of NKC and MHC class II genes within the Central and South American jaguar individuals, we observed that NKC variability surpassed that of MHC class II genes (Table 1). This aligns with prior research conducted in domestic cats, where NKC exhibited greater variability than MHC, encompassing class I, II, and III genes [51]. The number of MHC class II DRA alleles (*n* = 12) found in the 12 modern jaguars in this study was proportionally higher than the allelic diversity (*n* = 13) in DRB genes from 46 modern and historical African and Asiatic cheetahs [38]. Yet, previous studies on Namibian cheetahs did not find evidence of compromised immunocompetence even with low allelic diversity (*n* = 4 in 94 individuals) in MHC class II DRB genes [52]. Therefore, MHC gene diversity might be a poor indicator of population health in some wild felids. It is interesting to note that ungulates such as camels also exhibit a similarly low diversity in DRA (*n* = 3) and DRB (*n* = 5) alleles [53] without known reduction in their immunocompetence [54]. Furthermore, the diversity of TLR genes in the jaguar was high compared to those in the cheetah and African leopard [55], suggesting that jaguars might possess a heightened innate immunity, which could compensate for potential genetic depletion in certain adaptive immune genes [56].

Due to insufficient genome coverage, we could not adequately investigate the selected gene families within the Central American and South American lowland populations. Consequently, we could not contextualize the observed genetic diversity in the South American highland population within a broader framework. Missing a within-species comparison, the information gathered from the three gene families is limited and the immunome-wide variability might be a better approximation for the jaguar’s adaptability until future studies provide sophisticated distribution-wide data on these specific genes. Nonetheless, the immunome of the South American highland population was less variable than that of both other populations, which might already indicate a reduced adaptive potential, and stressed the urgency to further protect this jaguar population. 

## Conclusions

Our study highlighted geographic differentiation within jaguar populations to address questions concerning distinct genetic diversity and future adaptive capacities. In our investigation, we have identified noticeable separation within the species and provided insight into potential geographical differences in the immunome among the three identified populations. This underscores the importance of acknowledging and conserving geographic regions that show threatened genetic diversity and habitat-specific adaptations crucial for the Jaguar’s future adaptability. Although sample ages differ between the studied populations, the recent timing of population declines in evolutionary terms, together with the absence of divergent clustering among historical samples, suggests that age is unlikely to be the primary driver of the observed genetic structure, which more strongly reflects geographic origin. Although low-coverage sequencing introduces some uncertainty, our use of genotype likelihood–based methods together with strict filtering minimizes these effects and supports the robustness of the observed patterns. To clarify our understanding of current genetic diversity, especially in Central America and the South American lowland population, additional modern samples are crucial to future studies.

Given the importance of ESUs in conservation, we propose a re-assignment of the jaguar to facilitate targeted conservation efforts and propose the split between South and Central American population into different ESUs, and more in-depth analyses to investigate whether these could qualify for different subspecies. Additionally, it is imperative to identify and protect distinct jaguar populations harboring unique genetic diversity to prevent the loss of potential ecotypes, which could diminish the resilience and evolutionary potential of the entire species [57]. Conservation efforts must, therefore, take genetic diversities, especially those of immune response genes and population structure, into account to minimize genetic erosion and maximize adaptive potential [58]. Integrating population genomics and immune genetics into conservation strategies is essential for preserving genetic diversity, securing adaptability, and ensuring the long-term survival of jaguars and their corresponding ecosystems, ultimately safeguarding ecosystem function and human well-being [59].

## Methods

Our study aimed to investigate evolutionary history, genetic diversity and population structure of the jaguar by combining immune genetic and population genomic approaches. We analyzed genome-wide diversity and immune response genes to enhance our understanding of the species’ adaptability and to inform conservation efforts. The study was designed as a comparative genomic analysis utilizing contemporary and historical samples from various regions within the jaguar’s distribution. Samples included newly sequenced whole-genome data and existing datasets, incorporating museum specimens to provide an evolutionary context. This approach allowed us to assess genetic variation across different populations, focusing on immune genes such as TLR, NKC, and MHC class II, crucial for understanding disease resilience.

### Sampling

We used a combined set of 11 historical and 14 modern samples to investigate the population structure of jaguars. Modern samples collected after 1994 spanned 30 years preceding this study, while historical samples dated from 1853 to 1990. The data set generated in this study included two modern samples from the Brazilian state Mato Grosso do Sul and 11 historical samples from museum collections in Germany (Natural History Museums of Berlin, State Museum of Natural History Stuttgart, Senckenberg Naturmuseum, Alexander Koenig Research Museum), including three samples from Bolivia, two from the Brazilian Amazonas and one each from Mato Grosso do Sul, Costa Rica, Guatemala, Honduras, Paraguay, and Surinam (Fig. 1a, Additional file 2: Table S2). Additional, short-read data for 11 modern samples from Brazil and 1 sample from Mexico were obtained from a previous study [21] (NCBI BioProject: PRJNA348348, Additional file 2: Table S2). However, the data for an additional Guatemalan sample (MFGT) from the same study was not publicly available at the time of this study.

Based on the level I ecoregions of Central and South America [60], we categorized the samples into three populations based on the sample’s geographic origin: samples originating from North and Central America were assigned to the Central American population. Samples from the Amazonian (-Orinocan) lowland, Gran Chao, and Pampas were assigned to the South American lowland population, and samples from the Eastern highlands, including Cerrado, Caatinga, and Atlantic Forest, were considered members of the South American highland population. For samples PO31 and PO32, precise geographical locations were not available; only the Brazilian state of Mato Grosso do Sul was specified, and as this state predominantly comprises the Cerrado ecoregion, both individuals were assigned to the South American highland population.

### DNA extraction

Genomic DNA was extracted from jaguar samples, including dried skins and bones from museum collections, using a modified salting-out DNA extraction method [61]. Overnight, all samples were rehydrated in nuclease-free water to remove and dilute potential secondary preservatives before DNA extraction. Samples that failed the initial DNA extraction method were additionally prepared at the Ancient DNA laboratory at the University of Vienna (Austria) using a modified extraction protocol for historical samples [62]. To remove surface contamination, dried skin and bone samples were radiated for 30 min under UV-light, and skin samples were additionally bleached for 2 min using a 10% sodium hypochlorite solution. After washing with ddH_2_O and drying the samples, ~ 200 mg was ground using a Retsch MM 400 ball mill (Retsch GmbH, Haan, North Rhine Westphalia, Germany) and at least 50–100 mg powdered sample was used for DNA extraction [63]. All extraction batches included blanks to monitor cross-contamination during sample preparation, and extraction; no cross-contamination was detected, as evidenced by the failure of the indexing PCR amplification in these blanks during the final steps of NGS library preparation.

### Library preparation & sequencing

Illumina sequencing libraries for good quality samples (total DNA amount >20 ng) were prepared with the NEBNext^®^Ultra™ II DNA Library Prep Kit (New England Biolabs, Ipswich, Massachusetts, USA), while double stranded libraries for the samples extracted in the ancient DNA laboratory were prepared following the Meyer and Kircher protocol [64]. All libraries underwent paired-end sequencing on a Novaseq6000 platform (Illumina, San Diego, California, USA) at Novogene (Novogene company limited, Cambridge, UK)

### Genomic data processing

Detailed commands for the analyses can be found in the supplementary file. The short-read data of all individuals were trimmed using fastp v.0.20.1 [65] (RRID: SCR_016962) with base correction and low complexity filter enabled to remove sequencing adaptors, and polyG stretches at the end of the reads. We used a four-bp sliding window to detect regions of poor quality (Phred score < 15). Reads were removed if they fit into one of the following categories: reads below 36 bp length, reads with >40% low-quality bases, or reads with five or more undetermined bases (Ns).

Each sample’s trimmed reads were mapped to the chromosome-level jaguar assembly [66] (GCA_028533385.1) using bwa-mem v.0.7.17 [67] (RRID: SCR_010910). The resulting mapping files were sorted by assembly position, converted to BAM format, and indexed using SAMtools v.1.9 [68] (RRID: SCR_002105). Duplicate reads were identified with MarkDuplicates in Picard v.3.1.1 (RRID: SCR_006525, Broad Institute, Cambridge, Massetshusetts, USA), followed by INDEL realignment using the GATK v.3.8.1 [69] (RRID: SCR_001876) tools RealignerTargetCreator to identify target intervals and IndelRealigner to perform local realignment. After realignment, we removed all reads from the BAM files that were marked as either unmapped, secondary, QC failed, duplicate, or supplementary, using SAMtools, keeping only reads in proper pairs mapped to non-repetitive autosomal regions.

To identify the repetitive regions in the reference genome, we first masked all known repeats for ‘Felidae’ using RepeatMasker v.4.1.4 [70] (RRID: SCR_012954). Next, we generated a *de novo* repeat library with RepeatModeler v.2.13 [70] (RRID: SCR_015027), followed by another round of RepeatMasker to mask the remaining repeats. The masked regions of both RepeatMasker runs were combined to generate a regions file (BED) of non-repetitive regions on the 18 autosomes in BEDtools [71] v.2.31.0 (RRID: SCR_006646), which was used in the filtering of the BAM files.

Furthermore, we conducted a blastp v.2.15.0 (RRID: SCR_004870 [72]) search against the Swiss/UniProt database v.2024.02 [73], as part of the rGO2TR [74] pipeline to extract exon regions from the annotation of the jaguar reference genome according to the GO-terms associated with immune response (GO:0006955), adaptive immune response (GO:0002250), and innate immune response (GO:0045087), respectively. We then used the BED-files generated by rGO2TR to filter the whole genome BAM files in BEDtool v.2.31.0 [75] to keep only reads mapping in the respective target regions.

Detailed post-processing statistics for each individual jaguar sample are provided in the supplementary material (Additional file 2: Table S2).

### Inferring population structure & estimating effective migration surface (EEMS)

To evaluate the potential impact of post-mortem DNA damage, we used mapDamage v2.0 (RRID: SCR_001240 [76]) to quantify fragment misincorporations for all historical samples, confirming only low levels of terminal C→T and G→A substitutions (Additional file 1: Fig. S6A–M), which were accounted for by stringent filtering in downstream analyses. Genotype likelihoods were calculated from the whole genome BAM files using ANGSD v.0.940 with the following key parameters: -GL 1 (specifying the SAMtools genotype likelihood model), -doGlf 2 (outputting binary genotype likelihoods in Beagle format), -doMajorMinor 1 (inferring major and minor alleles), -doMaf 2 (calculating minor allele frequencies), -minMaf 0.02 (removing sited with minor allele frequencies lower 0.02), -SNP_pval 1e-6 (identifying variable sites at a significance threshold of 1e-6), -minQ 20 (filtering reads with base quality below 20), -only_proper_pairs 0 (including all paired reads, not just proper pairs), -noTrans 0 (including transversions and transitions), -minInd 3/4 (requiring data for at least 75% of individuals), -setMinDepth 5x (requiring a minimum sequencing depth of 5), and -doCounts 1 (enabling allele counts) [77] and further pruned for linkage disequilibrium (LD) with ngsLD v1.1.1 [78]. LD was estimated as r2 values for all SNP pairs up to 500 kbp apart. An LD decay curve was plotted for a random sample of 0.05% of all estimated r2 values, with a bin size of 250, to establish suitable thresholds for linkage pruning. All sites were pruned, assuming a maximum distance of 75 kbp between SNPs and r2 ≥ 0.1.

A covariance matrix was calculated from genotype likelihoods of 689,785 LD pruned SNPs using PCAngsd v.0.9757 [79] and used to perform a principal component analysis (PCA) with the default settings of the ‘prcomp’ function [80] in R v.3.6.0 [81]. Additionally, the pairwise fixation index (Fst) for each jaguar population was calculated using the realSFS function of ANGSD, excluding samples PO31 and PO32 due to a high likelihood of false population assignment. The input data for realSFS was generated using ANGSD with the following commands: -GL 1 -doSaf 1 (raw output site frequency spectrum) -doMajorMinor 1 -minQ 20 -noTrans 0 -only_proper_pairs 0. The signatures of admixture between the different jaguar populations were calculated using ngsAdmix v.31 (RRID: SCR_003208 [82]). We performed 100 replicates for all ngsAdmix runs ranging from k = 2 to k = 6. We summarized and visualized the results using CLUMPAK [83]. We assessed the model fit of each K value to the data using evalAdmix v.0.95 [84], and plotted mean likelihood values and their standard errors using R v.3.6.0 [85].

Additionally, we inferred effective migration rates between the different jaguar populations performing an EEMS analysis [85]. We performed three replicates with 1 million MCMC iterations each, using a burn-in of 100,000 and 7,000 demes. For some of the 25 Jaguar samples used in this analysis, the collection sites’ precise coordinates were unavailable. Consequently, we either assigned coordinates within the wider known sampling area or, in instances where sampling localities were unknown, utilized the coordinates of the center of the respective country or state.

We further reconstructed the phylogeny of the 25 jaguars using the phylogenetic network approach implemented into SplitsTree [86]. To do so, we called genotypes using ANGSD v.0.940 (flags: -GL 1 -doMaf 2 -minMaf 0.02 -doMajorMinor 1 -doGeno 2 -doPost 1 -doSaf 1 -fold 1 -SNP_pval 1e-6) and converted these to the adegenet input format using PopGenTools (https://github.com/CGRL-QB3-UCBerkeley/PopGenTools). Next, we calculated genetic distances using the R package adegenet [87]. Lastly, we visualized the phylogenetic network topology using Splitstree.

### Estimation of heterozygosity and inbreeding

Genome- and immunome-wide heterozygosity was estimated for all samples with adequate coverage (≥ 5x) based on the folded side frequency spectrum (SFS). The per-sample site allele frequencies were estimated with ANGSD v.0.940 (flag: -doSaf 1) using the reference genome as ancestral. BAQ2 computation (flag: -baq 2) and mapping quality adjustment were enabled. A minimum score of 30 was set for both mapping and base qualities, and a maximum depth cut-off was set to the 95th percentile of the sample’s depth distribution. The per sample folded SFSs were generated in realSFS (flag -fold 1) with 100 bootstrap replicates. Heterozygosity was then calculated in R v.3.6.0. as the percentage of heterozygous sites out of the total number of sites. For the whole data set, inbreeding was estimated using ngsF v.3 [84] implemented in angsd with 1,500 iterations based on 5,037,004 SNPs calculated using the following parameters: -GL 1, -doSaf 1 (calculate the Site Allele Frequency likelihood), -doMajorMinor 1, -minQ 20, -doGeno 32 (output genotype probabilities), -doMaf 2 (calculate Minor Allele Frequency from GLs), -minMaf 0.02, -doPost 1 (calculate posterior probabilities of genotypes), -doGlf 3 (output Beagle format), -noTrans 0, -only_proper_pairs 0, -SNP_pval 1e-6, -minInd 3/4, -setMinDepth 5x, -doCounts 1.

### Nucleotide diversity of 11 historical and 12 modern jaguars

To calculate nucleotide diversity, a vcf file was generated from genotype likelihoods of the LD-pruned SNPs with ANGSD v.0.930 (flags: -GL 1 -doGlf 2 -doMajorMinor 1 -dovcf 1 -doPost 1 -doMaf 1 -minMaf 0.02 --ignore-RG 0 -doGeno 1 -SNP_pval 1e-6 -minQ 20 -noTrans 0 -only_proper_pairs 0 -minInd 3/4 -setMinDepth 5x -doCounts 1) for the whole genome and immunome, and diversity statistics were calculated with the stacks populations tool v.1.32 [88] (flags: --fstats -k --smooth-fstats --smooth-popstats --bootstrap) for each population (Central American vs. South American lowland vs. South American highland) separately. This generated mean nucleotide diversity values across the genome and immunome, as well as per-site nucleotide diversity (equivalent to expected heterozygosity), which were then used for the statistical comparisons among populations. To assess differences in the distribution of nucleotide diversity across populations, we first tested for normality of residuals using the Anderson-Darling test with the nortest R package v.1.0–4 [89]. Due to significant deviations from normality in all datasets, we employed a non-parametric Kruskal-Wallis test [90] in base R v.3.6.0 (R Core Team, 2023). Subsequently, pairwise Wilcoxon rank sum tests were conducted with Benjamini-Hochberg correction for multiple comparisons [91]. Boxplots with jittered data points were generated using ggplot2 v.3.5.2 [92] and ggpubr v.0.6.1 [93] to visualize the distribution of nucleotide diversity (Additional file 1: Fig. S7). Samples PO31 and PO32 were excluded from the nucleotide diversity calculations due to a high likelihood of erroneous population assignment, which could potentially obscure genetic differences among populations.

### Immune response gene analyses

To explore the immune genetic diversity and understand the adaptive potential of the central, high- and lowland jaguar populations, we looked at single-copy genes from one adaptive (MHC class II) and two innate (TLR, NKC) immune response gene families. We mapped the short reads to the reference sequences of these genes derived either from the annotation of the jaguar assembly (GCF_028533385.1) or the cheetah assembly (GCA_027475565.2 [94]) using bwa-mem v.0.7.17 and removed PCR and optical duplicates with Picard v.3.1.1. Unmapped reads were removed, and the reads in the resulting mapping file were converted to a new fastq file using SAMtools view v.1.9. The filtered reads were then mapped a second time using bowtie2 v2.4.5 (RRID: SCR_016368 [95]) without any clipping (flags: -end-to-end -x -S) to avoid miss-called SNPs due to over-representation caused by falsely clipped reads. The variant calling of both alleles was performed with freebayes v.1.3.7 (RRID: SCR_010761 [96], flags: --report-monomorphic --skip-coverage 10), and written into a vcf file. We used bcftools consensus (RRID: SCR_005227 [97], flags: -H I) to create a consensus fasta file containing variable sites as ambiguity codes. The script used for mapping and variant calling is provided in the Supplementary material. Insertions and deletions (indels) were curated manually, and each called SNP was re-checked for validity by eye based on the mapping files using Tablet v1.21.02.08 [98]. A SNP was considered valid during manual curation if at least six independent reads covered the position of concern, and the minor allele was called if at least 33% of all reads supported the position.

We used the PHASE function implemented in DnaSP v.6.12.03 [99] to derive the alleles of the short-read consensus sequences with a threshold of 0.6, allowing for recombination. Heterozygosity [H], and haplotype diversity [h] were calculated in DnaSP using phased allele sequences.

## Supplementary Information

Supplementary Material 1.

Supplementary Material 2.

## Data Availability

All data generated and/or analyzed during this study have been submitted to public repositories: The raw sequencing reads are deposited as FASTQ files in Genbank under BioProject PRJNA1105330 (https:/www.ncbi.nlm.nih.gov/bioproject/PRJNA1105330). The immune response gene alignments for 13 modern jaguars are publicly available on the Phaidra repository under o:2917 (https:/phaidra.vetmeduni.ac.at/o:2917). All custom analysis scripts used in this study are publicly available on GitHub (https:/github.com/rmeissner95/jaguar-genomics) and archived on Zenodo (https:/doi.org/10.5281/zenodo.17513629). References for the datasets and code repositories are included in the reference list [100, 101].
