# Supplementary material for: Unraveling genome- and immunome-wide genetic diversity in modern and historical Jaguars
Source: Genome Biol. 2025 Dec 8;26:415. doi: 10.1186/s13059-025-03868-0 (PMC12683877; doi:10.1186/s13059-025-03868-0)
Supplement: Supplementary file 1 — Supplementary Material 1. [file 13059_2025_3868_MOESM1_ESM.docx]

**Supplementary figures**


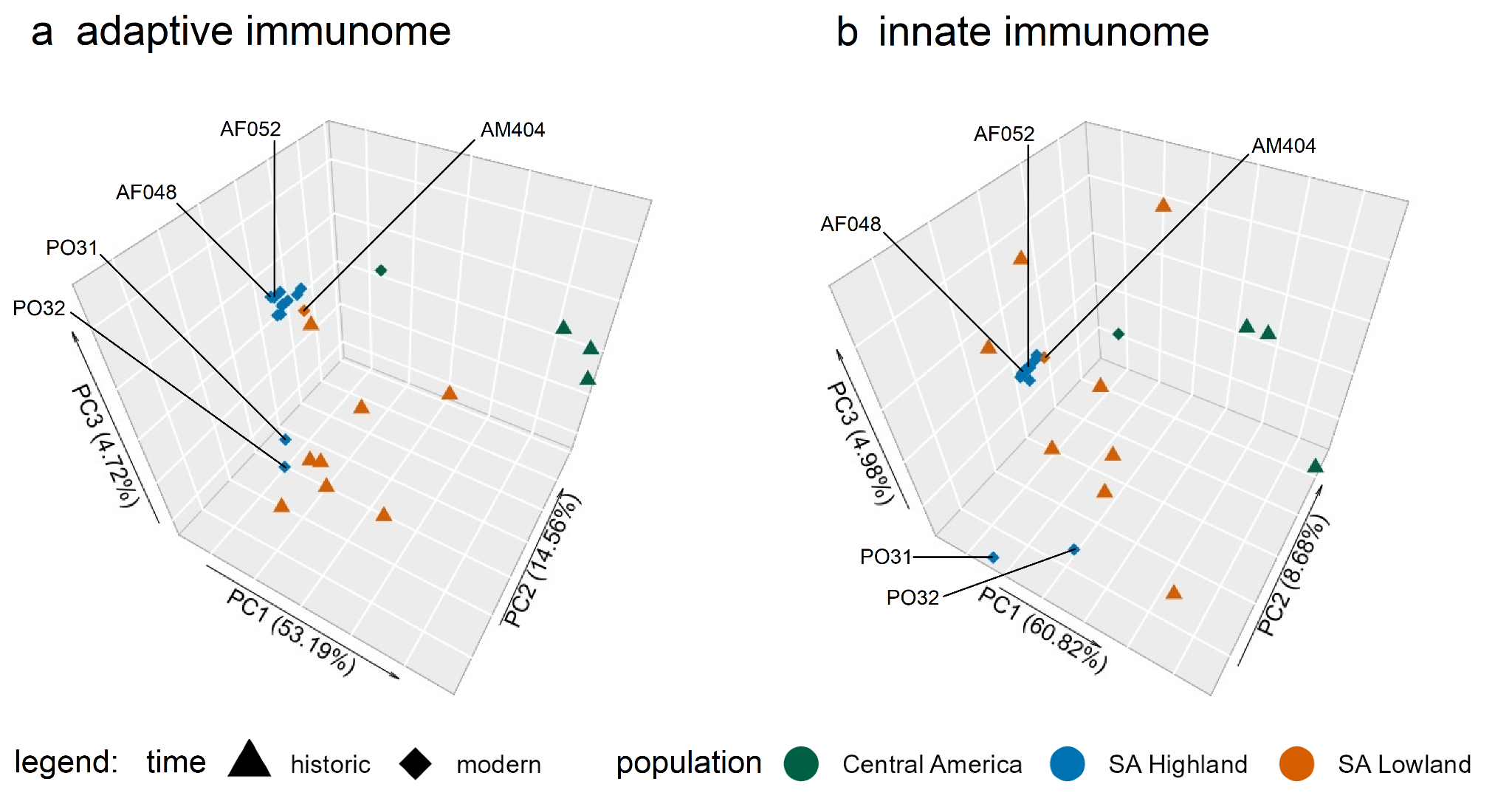


Fig. S1: Principal component analysis of 14,155 unlinked adaptive immunome-wide SNPs (a), and principal component analysis of 26,408 unlinked innate immunome-wide SNPs (b) of 25 jaguar individuals including the first three principal components.


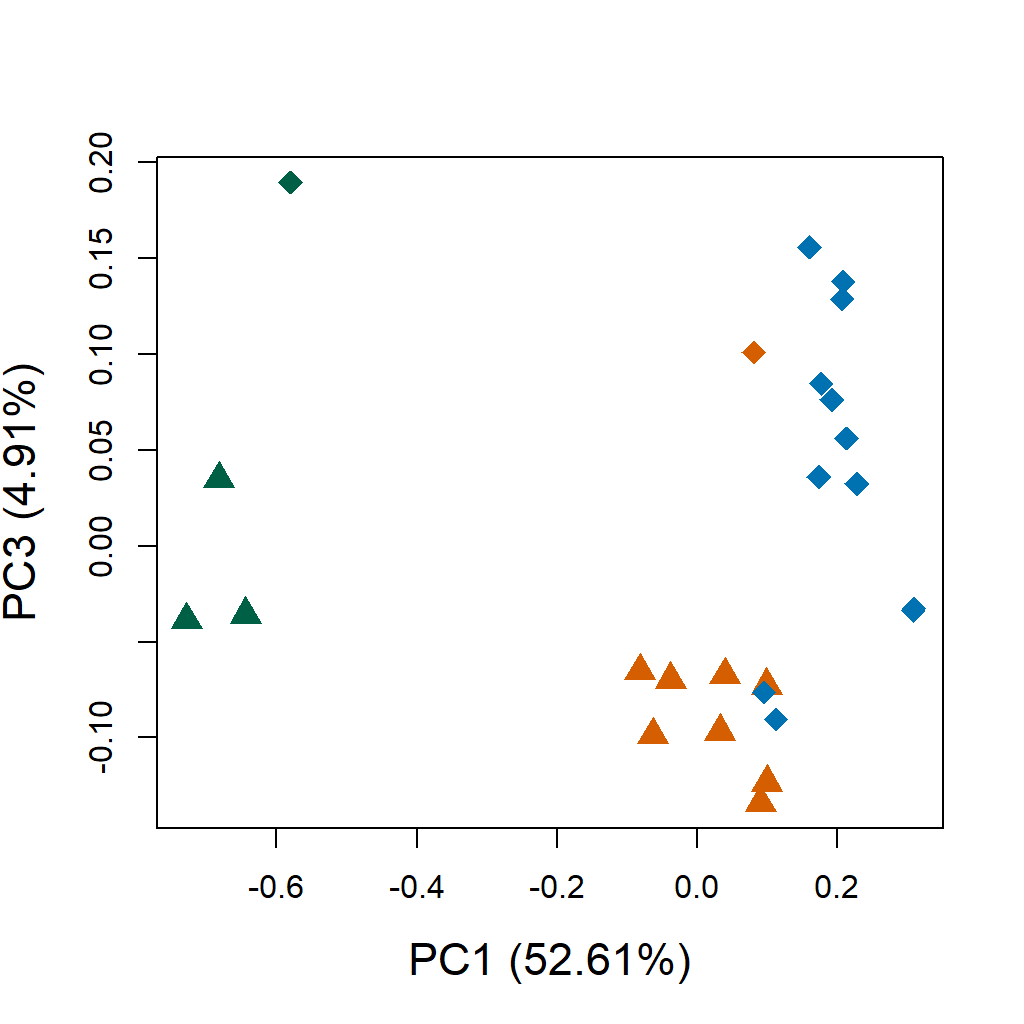


Fig. S2: PCA of 689,785 unlinked whole genome-wide SNPs of 25 jaguar individuals showing the first and third principal component, triangle represent historic samples, diamonds represent modern samples, green indicates samples from the Central American highland population, orange represents samples from the South American lowland population, blue represents samples from the South American highland population.


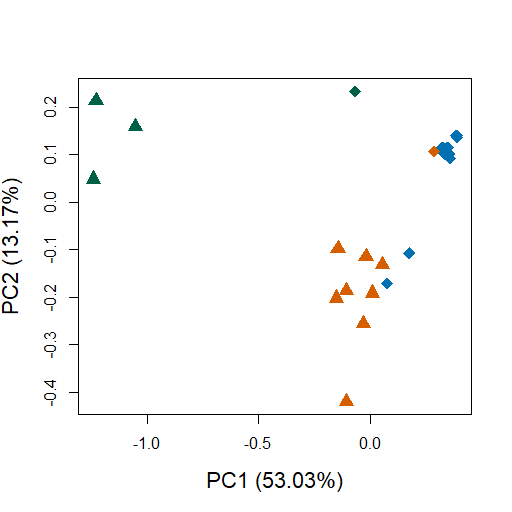


Fig. S3: PCA of 111,265 unlinked exome-wide SNPs of 25 jaguar individuals showing the first two principal components, triangle represent historic samples, diamonds represent modern samples, green indicates samples from the Central American highland population, orange represents samples from the South American lowland population, blue represents samples from the South American highland population.


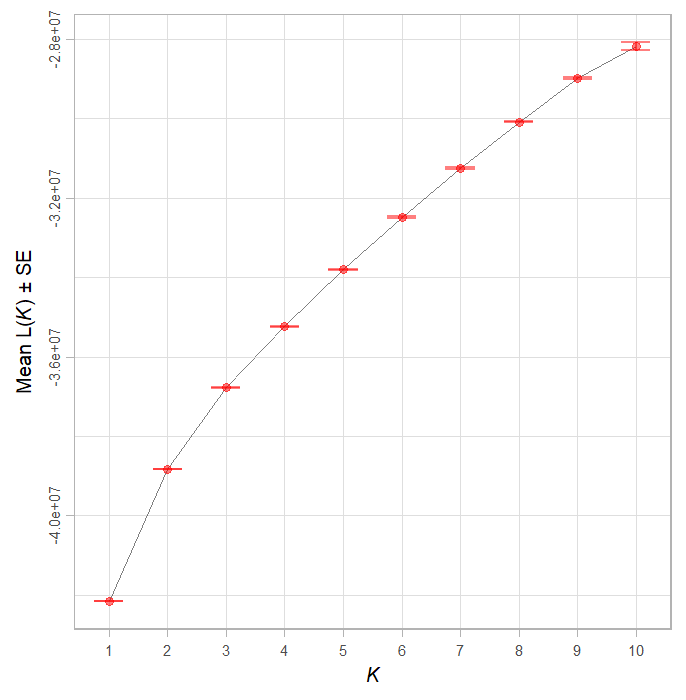


Fig. S4: Mean log likelihood of K (L(K)) ± standard error (SE) plotted against the number of assumed populations (K) for 25 Jaguar samples. The curve shows the average log likelihood values for each tested K, with error bars representing the standard errors.


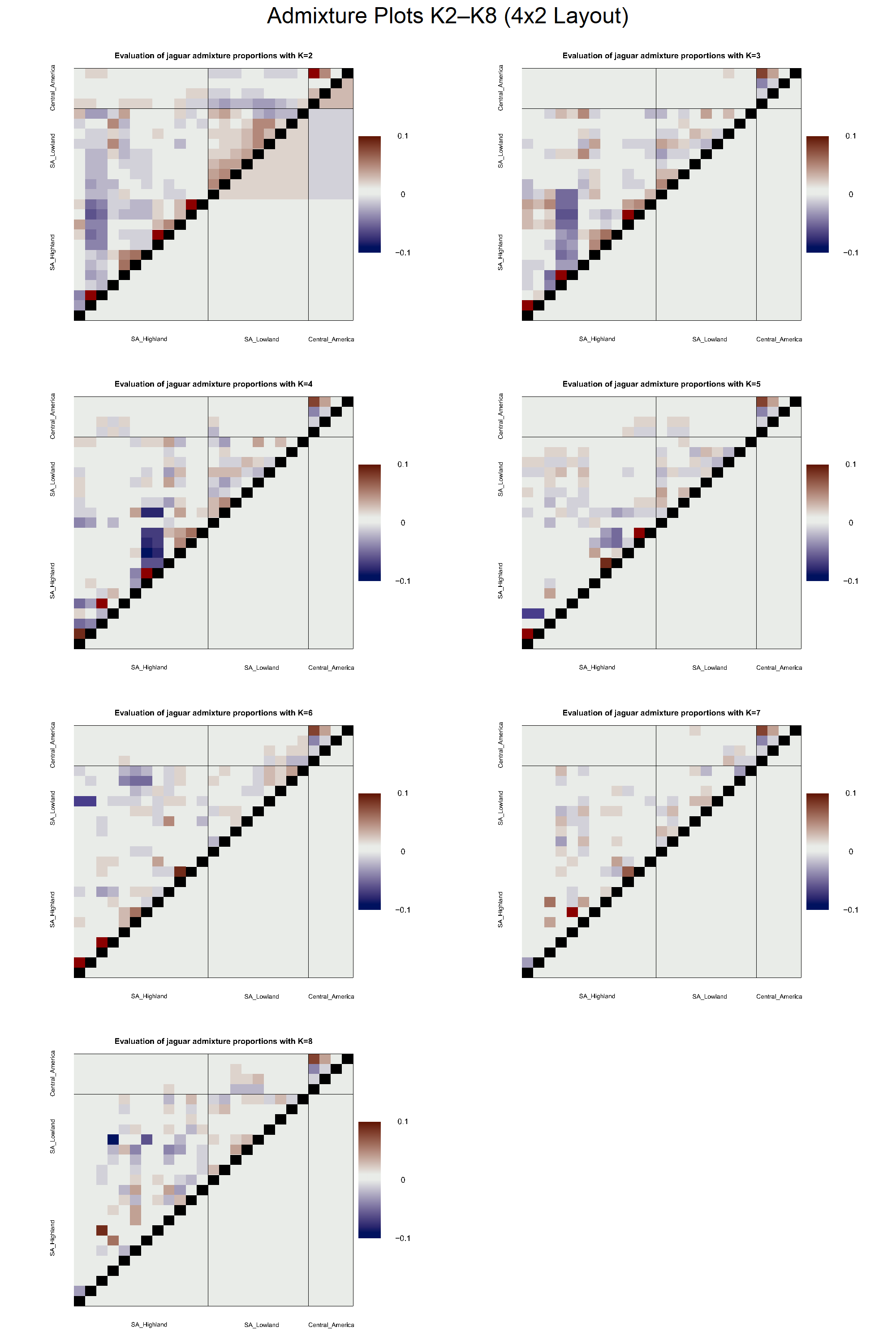


Fig. S5: Evaluation of jaguar admixture proportions for K = 2 to K = 8 using evalAdmix. Residual covariance matrices illustrate the fit between observed and predicted genotype ratio based on inferred admixture proportions. Positive (red) and negative (blue) residuals highlight deviations from model expectations. Population groups are separated by black lines. Lower residual signals indicate a better model fit.

A) PO04


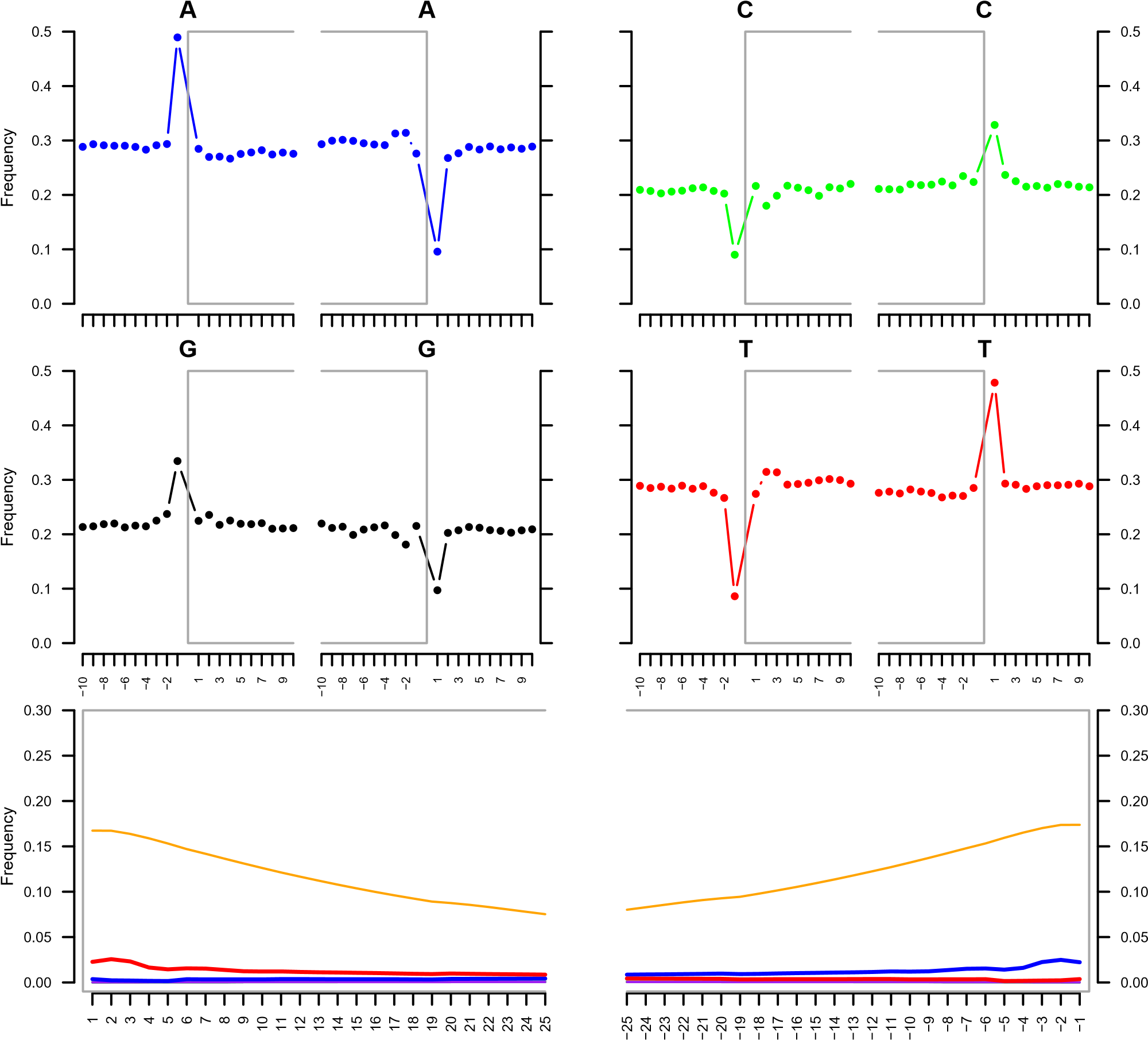


B) PO05


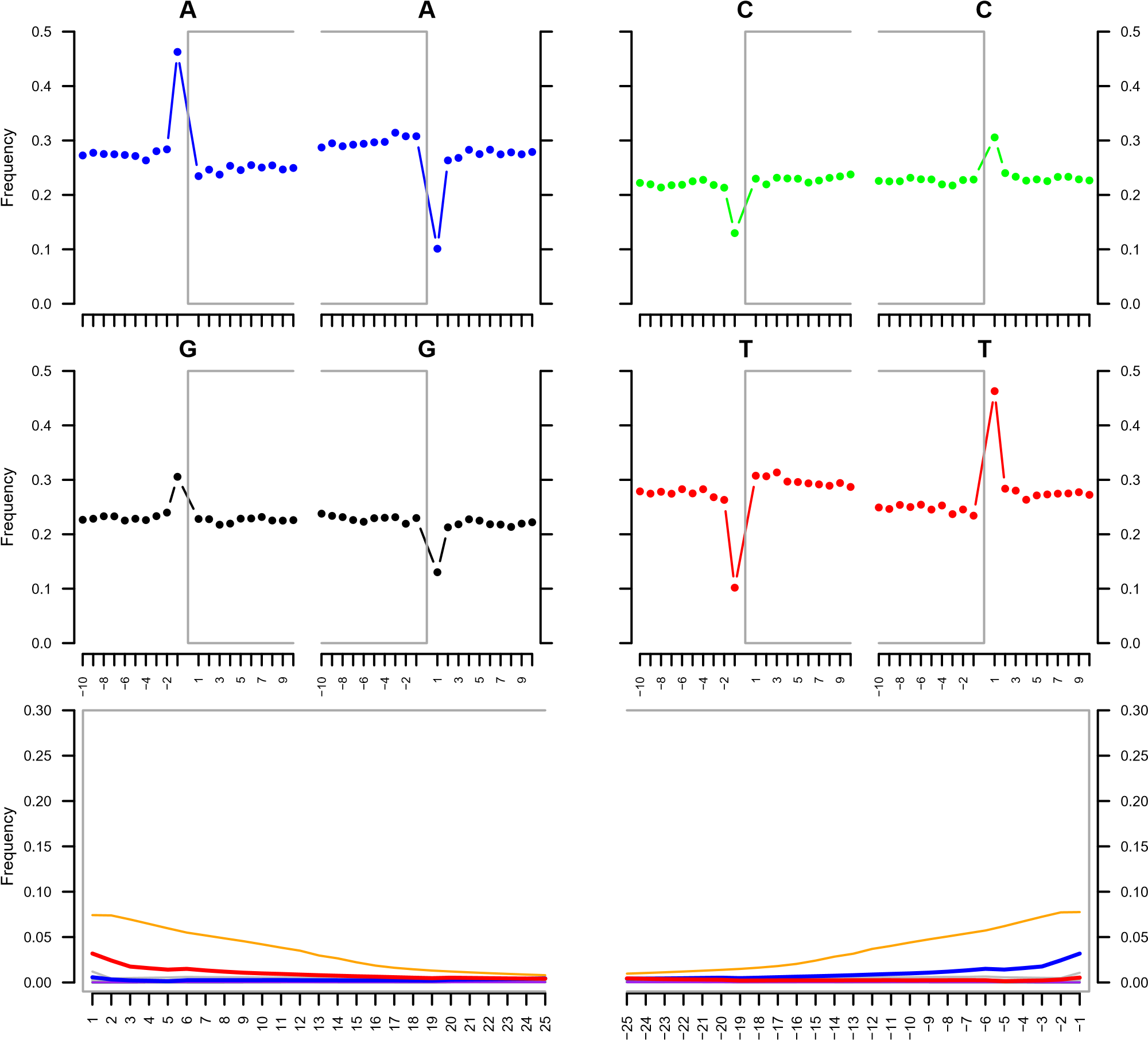


C) PO06


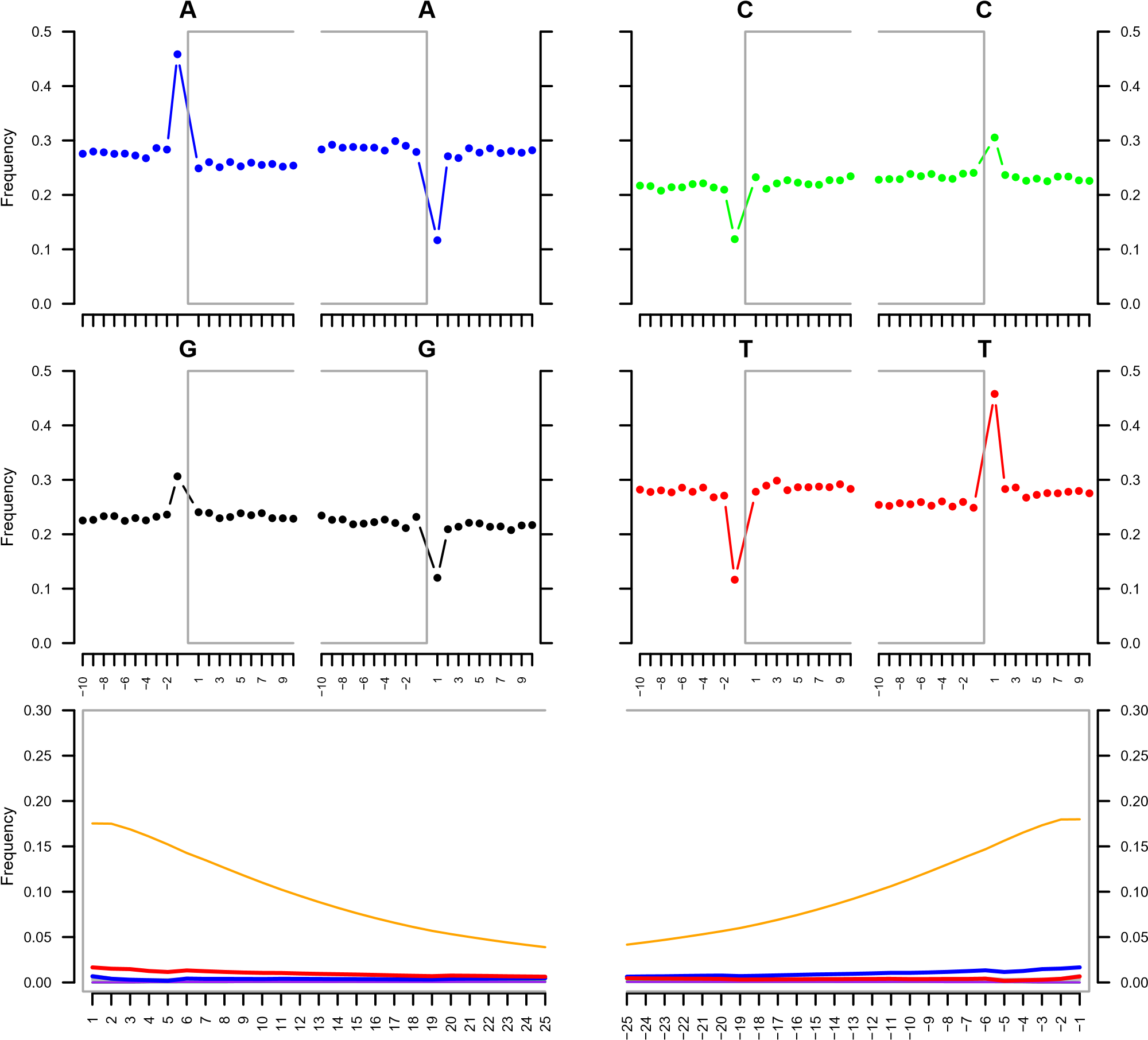


D) PO07


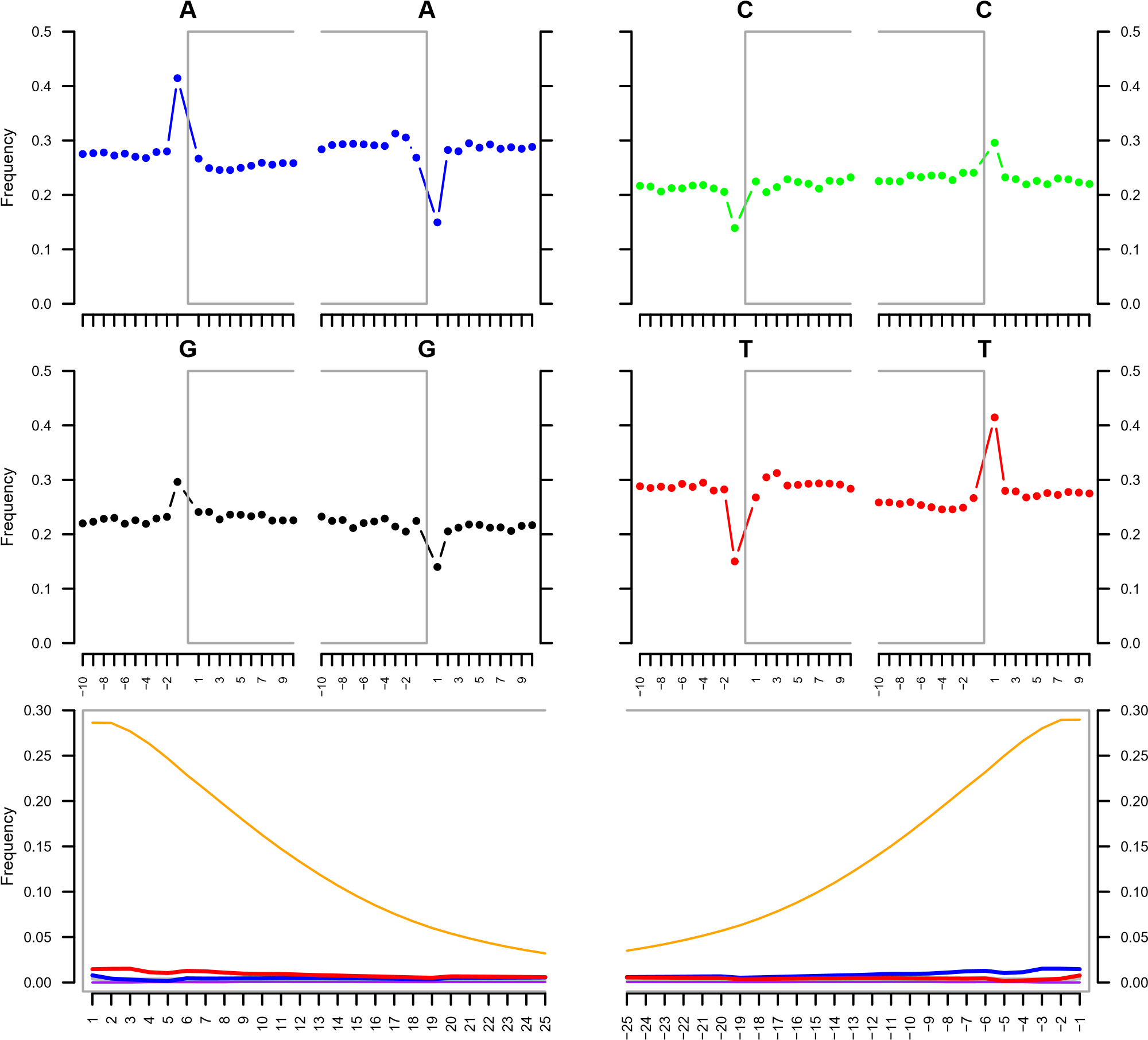


E) PO08


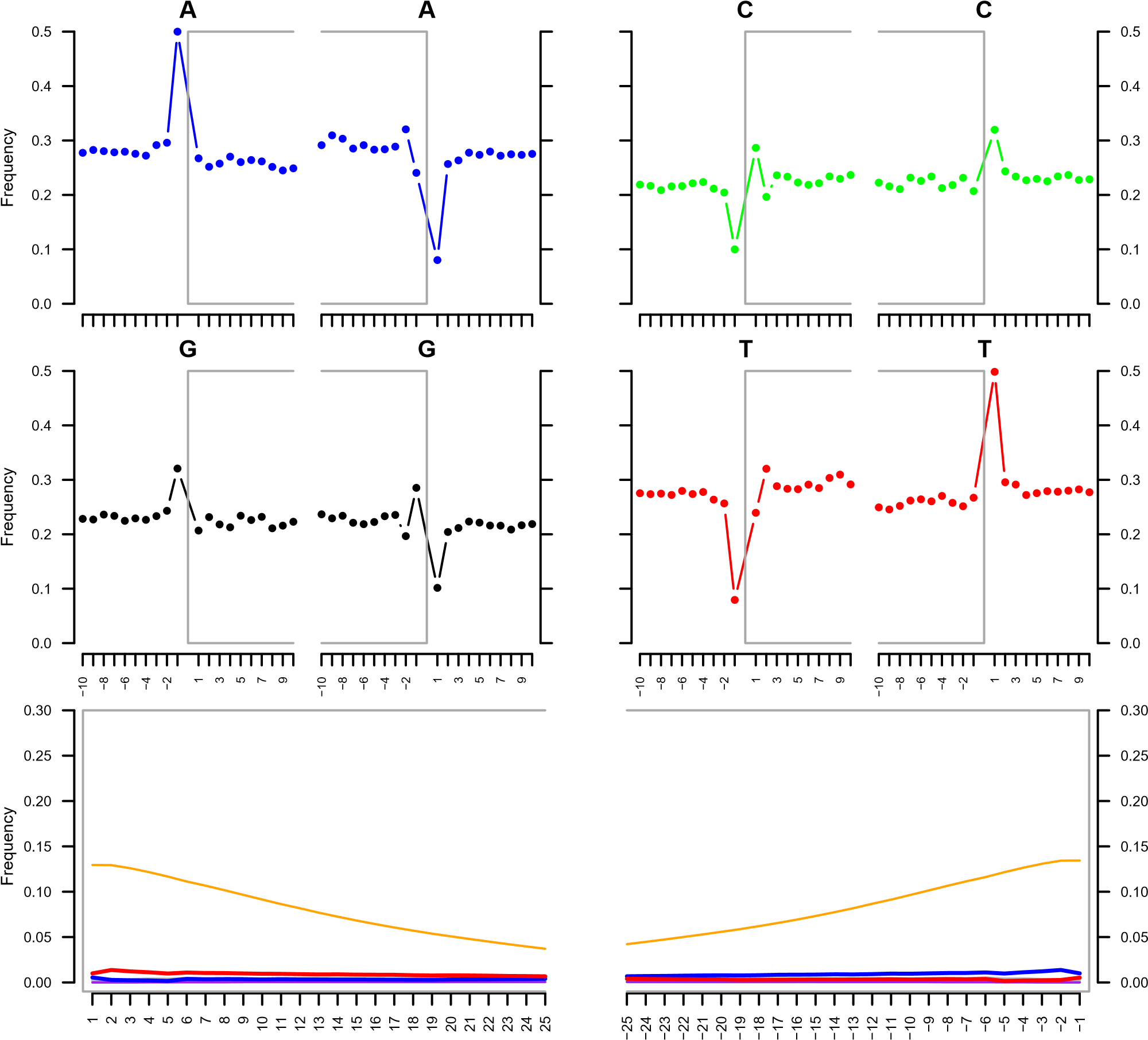


F) PO09


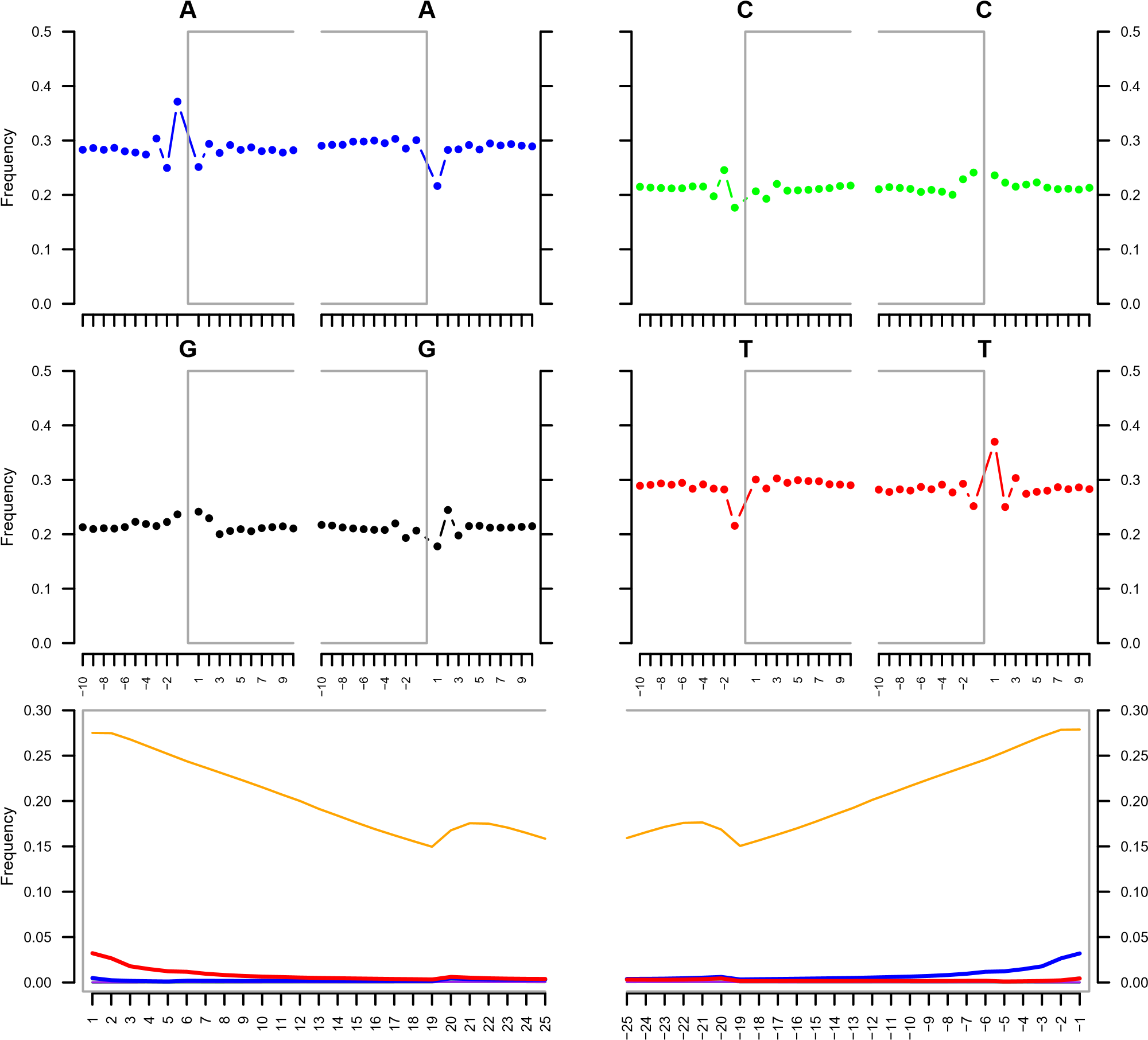


G) PO15


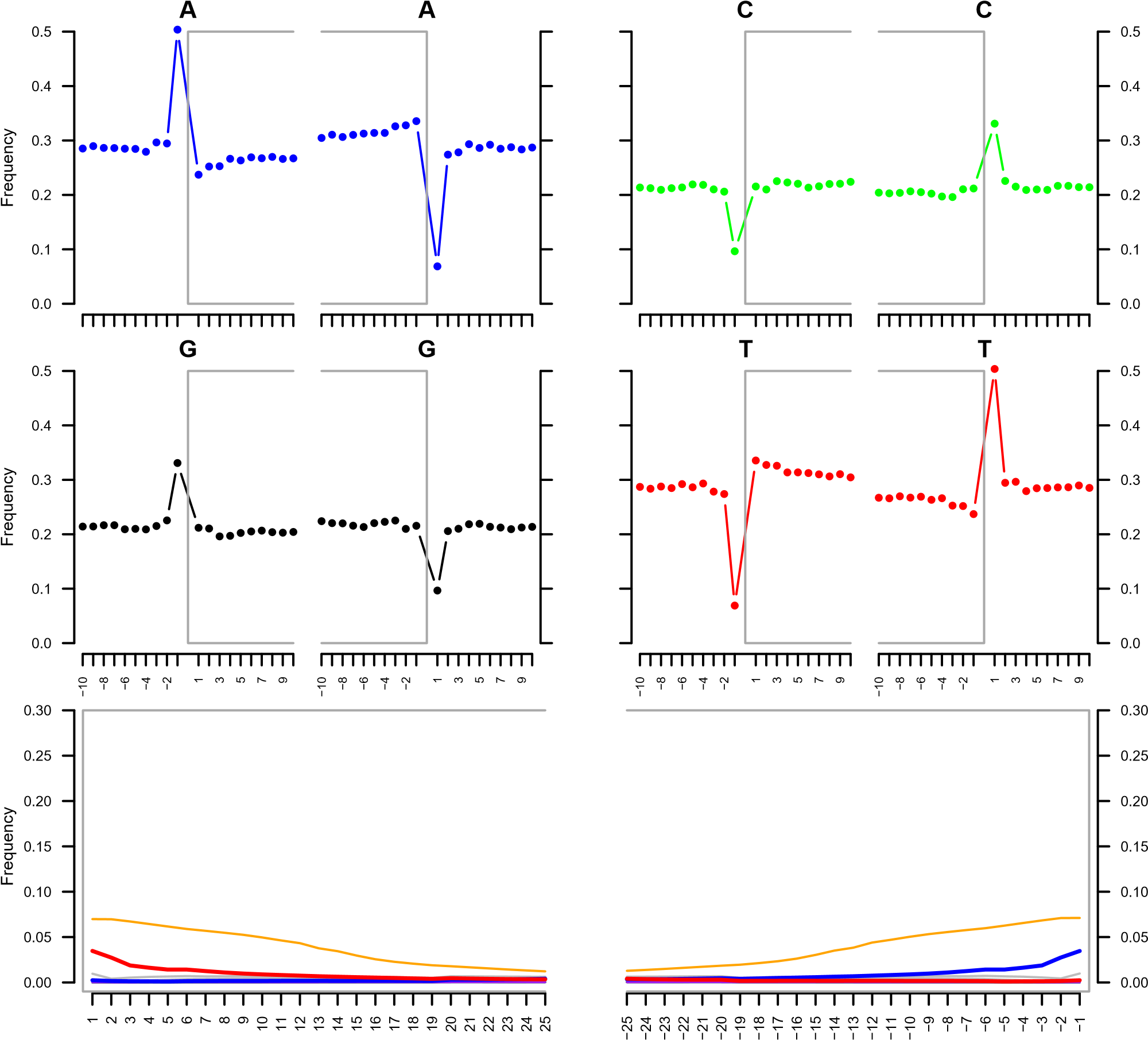


H) PO21


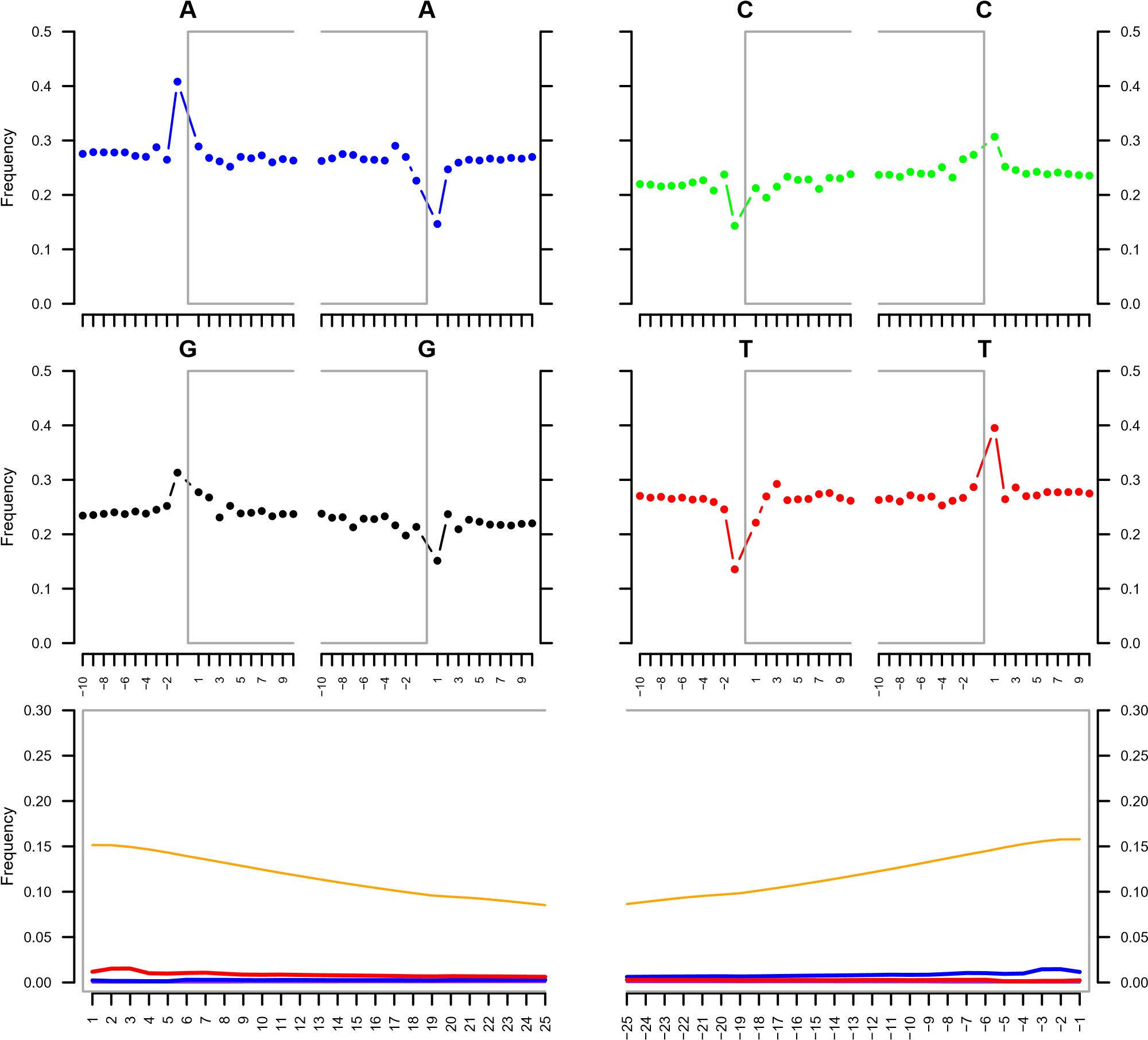


1. PO22


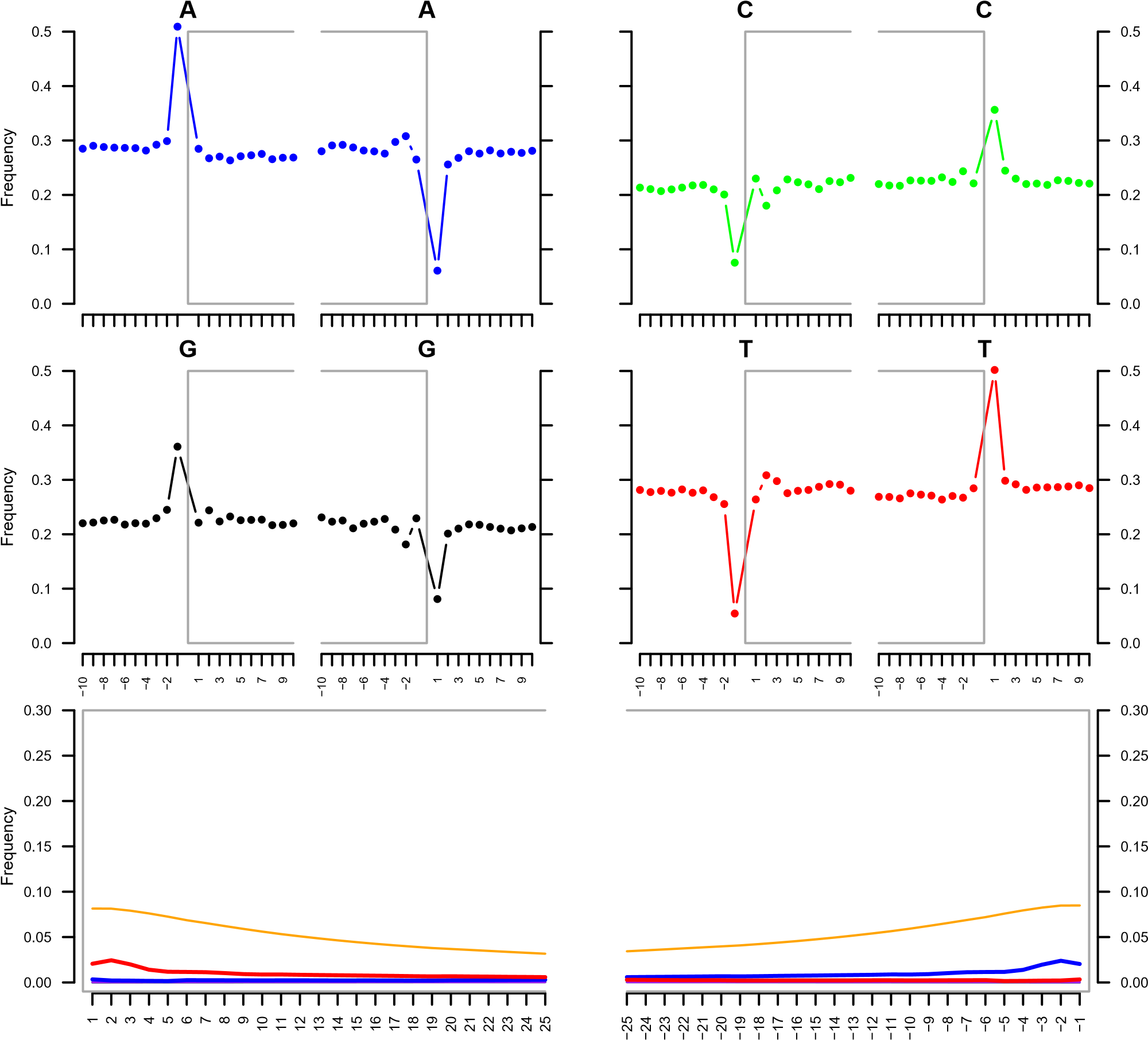


J) PO24


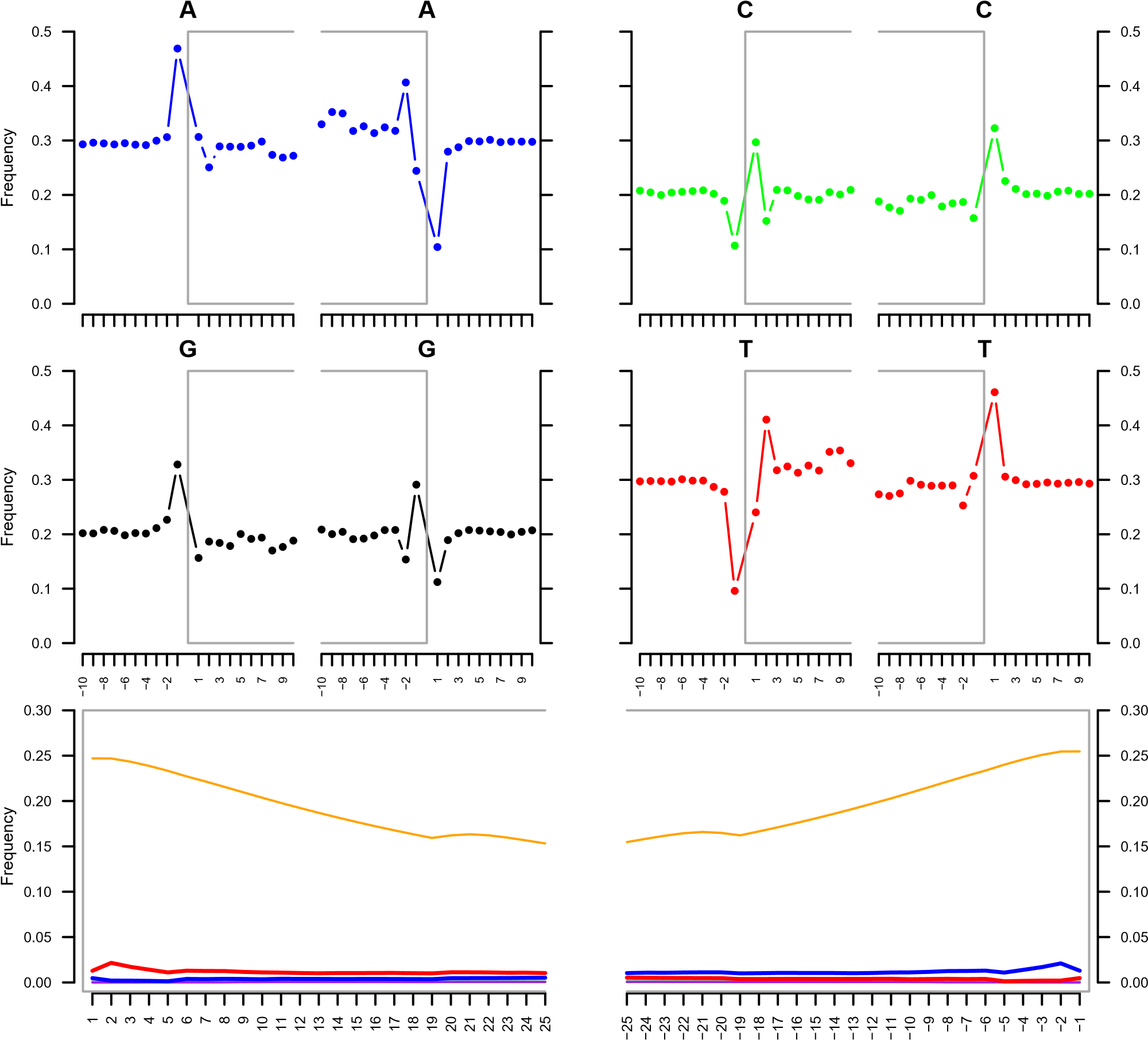


K) PO25


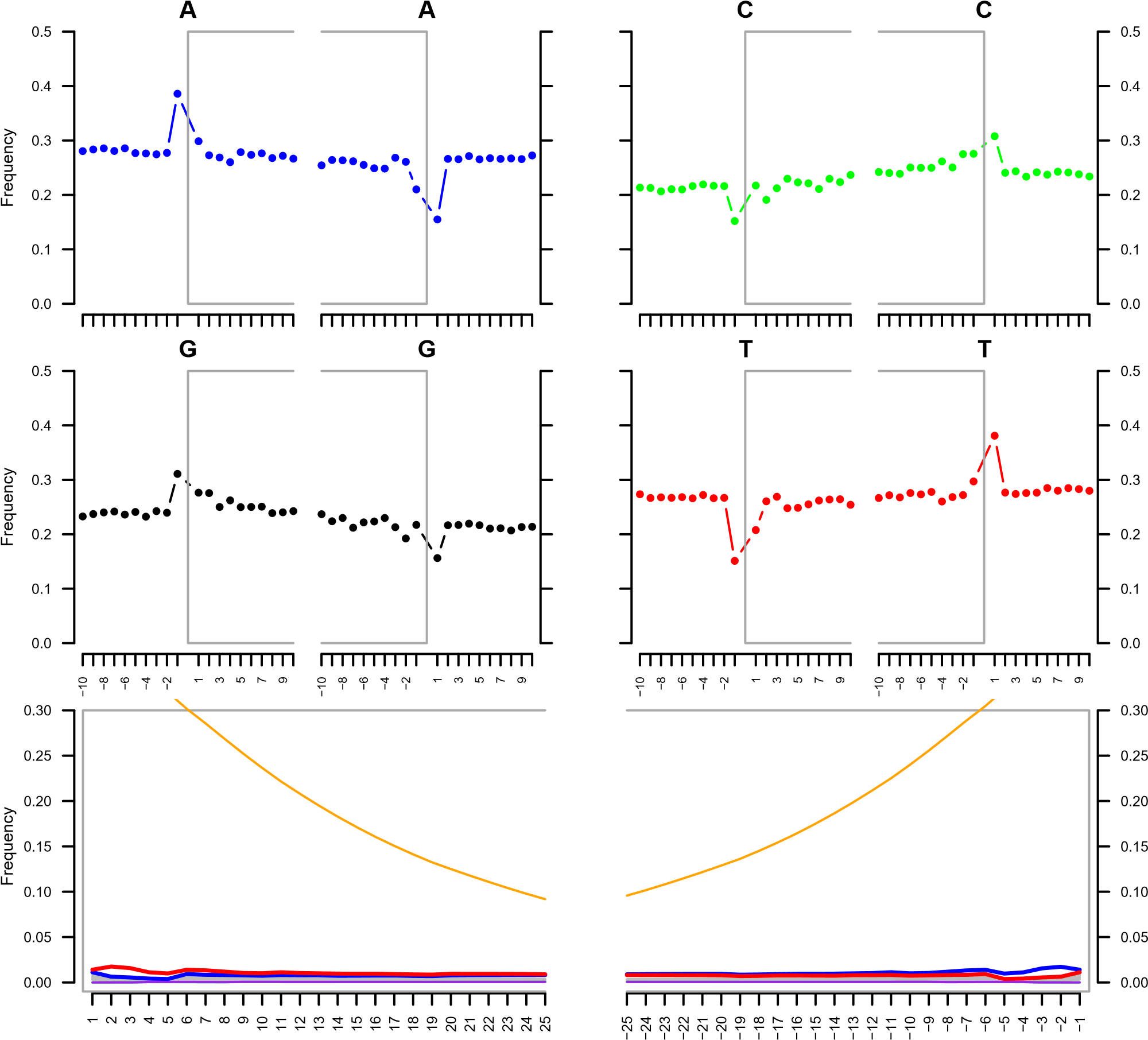


L) PO31


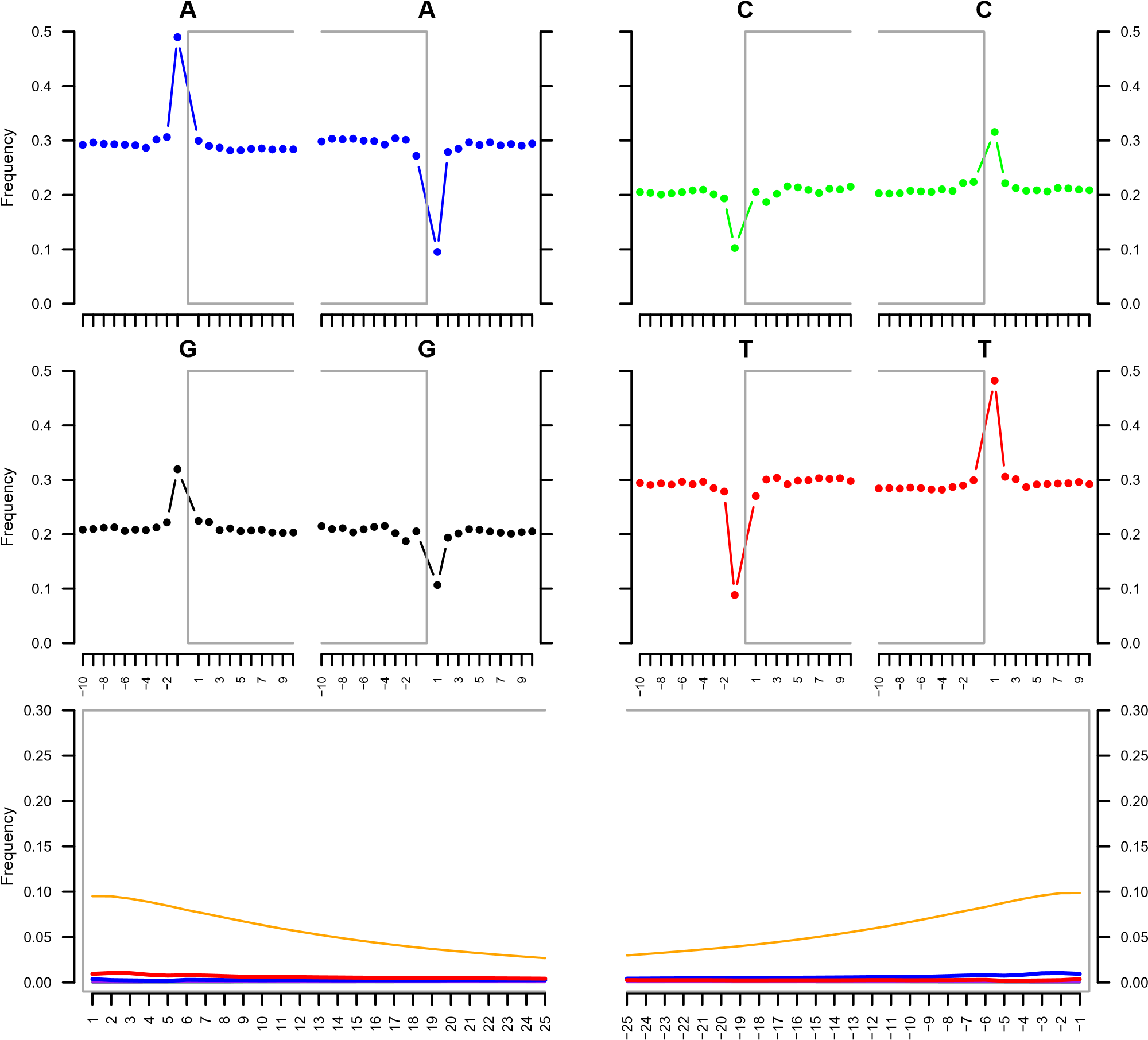


M) PO32


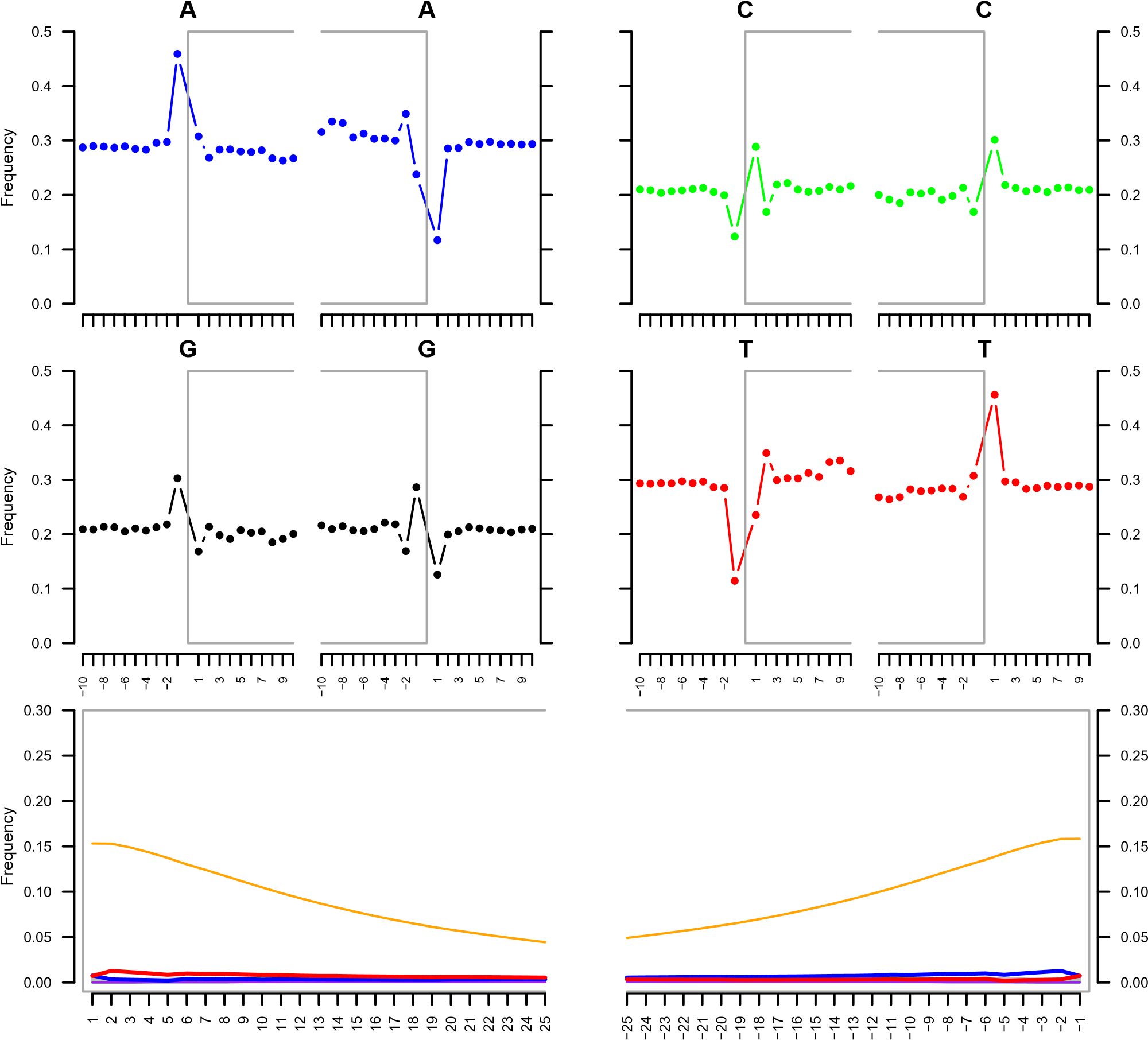


Fig. S6: Fragment misincorporation and nucleotide frequency patterns for 13 historical Jaguar samples (A-M), assessed using mapDamage. Each panel shows nucleotide frequencies at both 5′ and 3′ read termini (top four plots: A, C, G, T) and the position-specific frequency of cytosine deamination-induced misincorporations (C→T and G→A substitutions; bottom plots).


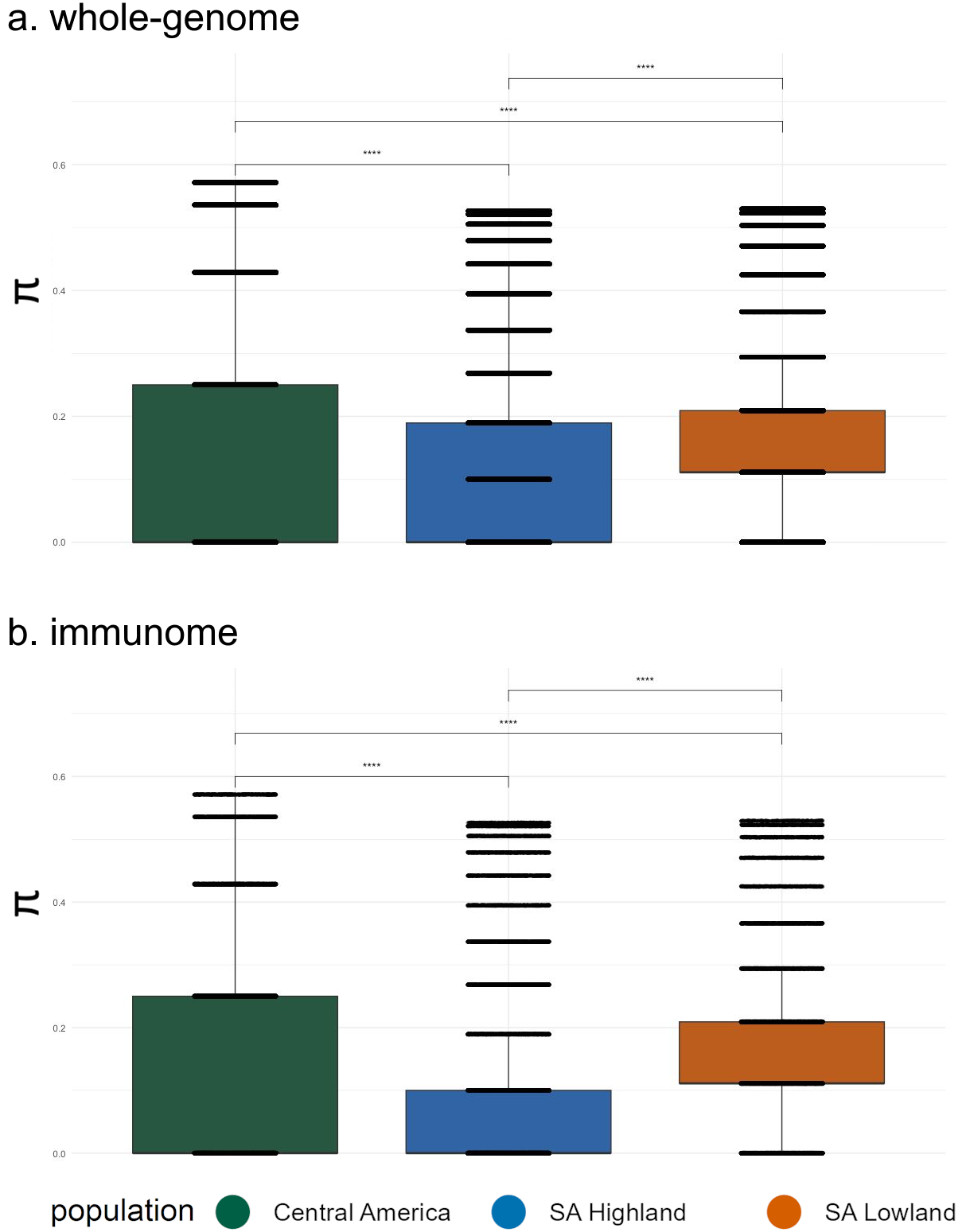


Fig. S7: Boxplots with jittered individual data points illustrating the distribution of per-site nucleotide diversity (π) (equivalent to expected heterozygosity) across populations for two datasets: (a) the whole-genome dataset and (b) the immunome dataset. Statistical significance of overall population differences was assessed using non-parametric Kruskal–Wallis tests (both p < 2.2 × 10⁻¹⁶). Subsequent pairwise Wilcoxon rank-sum tests identified the following significant differences between populations: Whole-genome dataset (panel a): Central America vs. SA Highland: p < 2 × 10⁻¹⁶, Central America vs. SA Lowland: p < 2 × 10⁻¹⁶, SA Highland vs. South America (Lowland): p < 2 × 10⁻¹⁶. Immunome dataset (panel b): Central America vs. SA Highland: p < 2 × 10⁻¹⁶, Central America vs. SA Lowland: p = 1.1 × 10⁻¹⁵, SA Highland vs. SA Lowland: p < 2 × 10⁻¹⁶.
